# Prepandemic psychotropic drug status in Portugal: a nationwide pharmacoepidemiological profile﻿

**DOI:** 10.1038/s41598-023-33765-0

**Published:** 2023-04-27

**Authors:** Luís Madeira, Guilherme Queiroz, Rui Henriques

**Affiliations:** 1 Instituto de Medicina Preventiva, Faculdade de Medicina da Universidade de Lisboa; Hospital CUF Descobertas, Lisbon, Portugal; 2 ACES Baixo Vouga, ARS Centro, Aveiro, Portugal; 3 INESC-ID and Instituto Superior Técnico, Universidade de Lisboa, Lisbon, Portugal

**Keywords:** Psychiatric disorders, Epidemiology, Public health

## Abstract

The prescription of psychotropic drugs has been rising in Europe over the last decade. This study provides a comprehensive profile of prepandemic consumption patterns of antidepressant, antipsychotic, and anxiolytic drugs in Portugal considering full nationwide psychotropic drug prescription and dispensing records (2016–2019) against several criteria, including active ingredient, sociodemographics, medical specialty, and incurred costs. An increase of 29.6% and 34.7% in the consumption of antipsychotics and antidepressants between 2016 and 2019 is highlighted, accompanied by an increase of 37M Eur in total expenditure (> 20M Eur in public copay) for these classes of drugs. Disparities in sociodemographic and geographical incidence are identified. Amongst other pivotal results, 64% of psychotropic drug prescriptions are undertaken by general practitioners, while only 21% undertaken by neurological and psychiatric specialties. Nationwide patterns of psychotropic drug prescription further reveal notable trends and determinants, establishing a reference point for cross-regional studies and being currently assessed at a national level to establish psychosocial initiatives and guidelines for medical practice and training.

## Introduction

Mental disorders are among the leading chronic non-communicable diseases in the world^1^, and Portugal is no exception, with an estimated prevalence of 18.4%. In 2019, psychological distress and depression in the Portuguese population reached 24% and 12.2% respectively, considerably higher than the European average, estimated at 11% and 7.2%, and of Spain, the neighbor country, estimated at 12% and 5.7%^2^. The commitment to deinstitutionalization policies and community-based mental health services have widened the access to psychotropic drugs and represents a step forward in mental health care. The prescription of psychotropic drugs, namely antidepressants, antipsychotics and anxiolytics, reported increasing trends in the last two decades both worldwide^3–7^ and in Portugal^8^, especially among women and the elderly^9,10^. However, thorough surveillance is important to tackle issues concerning inequalities in access, overuse of psychotropic drugs like benzodiazepines, and inadequate active ingredient selection^11^.

Portugal is a paradigmatic case of heavy use of anxiolytics since the 90s^12^, as this class represents two percent of all sold drugs, the highest consumption rate among the member countries of the Organisation for Economic Cooperation and Development (OECD)^2^. However, the prescription trend of this therapeutic group has remained stable^13^. As for antidepressants, Portugal is the second OECD country with the highest consumption rate of antidepressants, after a threefold increase from 2000 to 2020^12^. Finally, antipsychotic consumption has also registered considerable upward trends, registering 2012 levels similar to those of north-European countries^14^. However, most data on psychotropic drug prescription and consumption is outdated, especially concerning antipsychotics, and insufficient to comprehend the dynamics of access to mental healthcare, particularly the specialty responsible for that care, medication adherence, sociodemographic determinants, and disaggregated statistics by active ingredient. This information is vital to health policy and planning, and can help designing more effective and tactical campaigns aiming to improve the quality of prescription, the communication between primary and hospital care, and non-pharmacological support.

Most health data in Portugal is currently fully digital and centralized in the Shared Services Ministry of Health (SPMS), comprehending data on clinical records and an electronic prescription platform (PEM) that allows the tracing of every prescription, both from public and private sectors. This represents an excellent opportunity in the European context to study nationwide pharmacoepidemiological factors and offer a systematic assessment and characterization of the prescription and consumption trends of psychotropic drugs along the prepandemic period (2016–2019).

The proposed Portuguese psychopharmacoepidemiologic study aims at answering three major research questions: What is the Portuguese prepandemic status on psychotropic drug prescription and consumption? How is prescription activity distributed across medical specialties? What is the volume of associated expenditures? Complementarily, we further inquiry aspects of medication adherence and trends of prevalence–obsolescence per active ingredient. As a result, this study comprehensively reveals significant psychopharmacoepidemiologic trends, along with notable sociodemographic and geographic determinants, prescription prevalence per medical specialty, and total and copay expenditures. The acquired results offer an actionable map that can guide the subsequent establishment of public health initiatives.

## Methods

### Data collection

We performed a descriptive non-comparative cohort study, with data related to *all* citizens in Portugal with registered prescriptions of any antipsychotic, antidepressant, or benzodiazepine approved for commercial usage by Infarmed from 1/1/2016 to 31/12/2019. The list of available active ingredients per psychotropic drug class in Portuguese territory, together with their commercial packaging information, are listed in Supplement B.

Complementarily to prescription activity, the targeted cohort study further monitors every dispensation act at all pharmacies. Drug dispensing is used as the proxy to assess drug *consumption*. Drug *adherence* is subsequently defined as the ratio of dispensed drugs (in DIDs) against prescribed drugs (in DIDs). Citizens with active prescriptions during this period are referred to as patients, with the patient volume for a given drug being the number of patients with an active prescription of that drug.

For each *patient* (granted anonymity), we collected data on gender, age group (10-year ranges), primary care visits, received prescriptions, and undertaken dispensation acts. For each *prescription*, we collected the medical prescription identifier, the medical specialty of the prescriber, the week of prescription, the municipality where it took place, the package code (with information on active ingredient, commercial name, number of package units, dosage form and pharmaceutical formulation), and the quantity of prescribed packages. The term *active ingredient* is used in this study in reference to the underlying principle or substance of an individual drug. In addition, for each *dispensing* act at a pharmacy, we further collected the fraction of prescribed packages that were acquired, the week and municipality of the act, the total expenditure, and the governmental co-payment (subsidy). The total expenditure is defined by the applicable pricing at the pharmacy at the time of the dispensing act; whereas the governmental co-payment is defined as the eligible cost reduction over the total expenditure, a subsidization that is inherently patient- and drug-specific.

.

### Data analysis

Descriptive statistical analysis of the above psychopharmacoepidemiologic data was undertaken to retrieve notable time trends in consumption, prescription, adherence, and expenditure by a range of different sociodemographic variables, including patient’s age, gender, and residence.

The data records pertaining to drug and patient information, as well as prescription and dispensation acts, were mapped onto a relational database where the patient profile, geographical details, taxonomical drug information, and packaging details were decoupled from prescription-dispensing information to promote time and memory efficiency associated with data exploration tasks. Data were preprocessed to correctly map equivalent coding.

The patients’ municipality of residence was inferred from their primary health care centre, not from the place of prescription since it is often carried out in central hospitals.

Drug prescription rates are expressed in Defined Daily Dose per 1000 inhabitants-days (DID). The Defined Daily Dose (DDD) corresponds to the assumed average maintenance dose per day for a drug used for its main indication in adults. For this, we first assigned a DDD to each active ingredient in the study according to its Anatomical Therapeutic Chemical (ATC) code, using the guidelines of the World Health Organization (WHO)^15^. In the case of drugs without an ATC code, a DDD value was assigned based on previous studies with the same active ingredient (https://www.whocc.no/atc_ddd_index/, accessed 4/2023). The DDD units per package were calculated and assigned to their identifier. The complete list of packages and correspondent DDD can be consulted in Appendix B2. DID of each active ingredient was then obtained by multiplying the DDD units per package by its total prescription and divided by the target population size and period of study.

To standardize results by demography, the number of residents per municipality and district for different gender and age groups were collected from the portal Instituto Nacional de Estatística (INE). To account for meaningful deviations against national demographics, the geographic distribution of DID statistics was standardized by the age distribution of citizens along the Portuguese territory, unless stated otherwise.

Differences on the prescription or consumption rates between active ingredients and sociodemographic features were statistically assessed using *t*-test with a significance level of 0.001 if estimates pass the Shapiro–Wilko normality testing at 0.01 significance, otherwise the alternative non-parametric Wilcoxon paired test is applied.

The data processing, patient location estimates, and subsequent analytical tasks were conducted using Python and PostgreSQL. The graphical presentation of statistics was carried out in Plotly.

### Ethical approval

 Ethical approval granted by Ethics Committee of Centro Académico de Medicina de Lisboa (CAML) with reference number 340/20. The authors further declare full compliance with ethical regulations, including those principles embodied in the Declaration of Helsinki.

## Results

### Birds-eye view of data

This study covers a total of 46,161,485 prescriptions, of which 17,529,112 correspond to antidepressants, 6,541,283 to antipsychotics, and 22,091,090 to benzodiazepines. Tables 1, 2, 3 and 4 summarize the yearly statistics of the national cohort across drug classes, gender, age, and geography. Table 1 presents the distribution of consumption rates in DIDs; Table 2 assesses the adherence rate (consumption to prescription DIDs ratio); Table 3 provides the number of patients with active prescriptions; and Table 4 summarizes the total expenditures.

**Table 1:** Summary of the psychotropic drug consumption status in Portugal: consumption rates across demographic variables expressed in Defined Daily Dose per 1000 inhabitants-days (DIDs).

	Antipsychotics				Antidepressants				Benzodiazepines			
	2016	2017	2018	2019	2016	2017	2018	2019	2016	2017	2018	2019
Consumption rates (DIDs)												
All	10.93	12.90	13.38	14.17	83.03	97.78	103.66	111.82	57.86	65.31	65.00	64.33
Gender												
F	10.18	12.06	12.59	13.31	121.43	142.30	150.37	161.69	77.22	86.91	86.14	85.00
M	11.76	13.84	14.24	15.11	40.65	48.63	52.10	56.78	36.49	41.46	41.65	41.52
Age												
[18–29]	5.02	5.89	6.23	6.75	17.24	20.25	21.88	24.76	6.20	6.80	6.68	6.69
[30–39]	9.37	10.49	10.26	10.52	42.02	45.94	45.91	47.46	20.91	21.86	20.59	19.56
[40–49]	14.65	16.94	17.10	17.84	81.68	94.18	97.93	103.97	49.08	54.10	52.97	51.80
[50–59]	16.68	19.69	20.26	21.45	118.94	137.33	142.78	151.99	81.79	91.79	90.83	89.13
[60–69]	15.08	18.06	18.93	20.24	154.83	181.15	191.92	205.38	114.65	129.98	129.26	127.30
[70–79]	14.53	17.41	18.62	19.80	184.41	220.94	237.61	259.98	140.91	159.64	160.08	159.77
[+80]	22.25	27.88	30.35	32.86	195.68	248.02	275.85	306.71	162.92	191.37	195.63	198.28
Region												
Alentejo	10.83	13.18	13.82	14.63	78.33	91.22	96.81	102.85	45.26	50.06	48.80	47.92
Algarve	10.08	11.54	12.40	13.62	50.54	59.59	64.21	70.13	35.11	40.42	41.25	42.33
Centro	12.10	13.96	14.50	15.26	87.59	100.60	105.89	113.70	62.56	70.01	69.76	68.56
Lisboa	11.54	14.02	14.58	15.29	79.77	96.10	102.28	110.61	42.86	49.11	48.66	48.42
Norte	9.92	11.58	11.89	12.73	87.42	103.27	109.64	118.78	72.01	81.30	81.22	80.49

**Table 2:** Prescription adherence in Portugal: consumption to prescription DIDs ratio.

	Antipsychotics				Antidepressants				Benzodiazepines			
	2016	2017	2018	2019	2016	2017	2018	2019	2016	2017	2018	2019
Adherence ratio												
All	0.88	0.82	0.81	0.81	0.87	0.79	0.79	0.79	0.97	0.96	0.96	0.96
Gender												
F	0.87	0.80	0.80	0.80	0.86	0.79	0.79	0.79	0.97	0.96	0.96	0.96
M	0.88	0.83	0.82	0.82	0.87	0.80	0.79	0.79	0.97	0.97	0.96	0.96
Age												
[18–29]	0.86	0.80	0.79	0.78	0.86	0.77	0.76	0.76	0.96	0.95	0.95	0.94
[30–39]	0.87	0.81	0.80	0.80	0.86	0.77	0.77	0.77	0.97	0.95	0.95	0.95
[40–49]	0.87	0.82	0.81	0.80	0.86	0.78	0.78	0.78	0.97	0.95	0.95	0.95
[50–59]	0.87	0.81	0.81	0.80	0.85	0.78	0.78	0.78	0.97	0.96	0.96	0.95
[60–69]	0.88	0.82	0.81	0.81	0.87	0.80	0.80	0.80	0.97	0.96	0.96	0.96
[70–79]	0.88	0.82	0.82	0.81	0.87	0.81	0.80	0.80	0.98	0.97	0.97	0.97
[+80]	0.90	0.83	0.83	0.82	0.89	0.82	0.81	0.81	0.98	0.97	0.96	0.96
Region												
Alentejo	0.85	0.79	0.77	0.78	0.84	0.78	0.77	0.77	0.96	0.95	0.95	0.95
Algarve	0.87	0.78	0.78	0.77	0.85	0.74	0.75	0.75	0.97	0.95	0.95	0.95
Centro	0.87	0.82	0.82	0.81	0.86	0.79	0.80	0.80	0.97	0.96	0.96	0.96
Lisboa	0.87	0.80	0.79	0.79	0.86	0.78	0.77	0.78	0.97	0.96	0.96	0.95
Norte	0.89	0.84	0.84	0.83	0.88	0.81	0.81	0.81	0.97	0.97	0.97	0.96

**Table 3:** Number of patients with an active prescription of psychotropic drugs per demographic group.

	Antipsychotics				Antidepressants				Benzodiazepines			
	2016	2017	2018	2019	2016	2017	2018	2019	2016	2017	2018	2019
Number of patients												
All	388,305	417,156	432,809	446,390	1221,550	1,305,811	1,358,035	1,421,671	1,506,424	1561,381	1537,151	1523,839
Gender												
F	238,947	256,735	266,587	274,631	911,818	969,431	1,004,136	1,046,860	1,041,085	1073,270	1053,174	1041,837
M	149,358	160,421	166,222	171,759	309,732	336,380	353,899	374,811	465,339	488,111	483,977	482,002
Age												
[18–29]	19,853	21,398	22,186	23,546	54,576	59,380	61,886	67,373	54,585	57,737	58,612	59,670
[30–39]	32,060	32,222	31,393	31,175	107,118	107,940	106,091	107,395	110,312	110,130	104,147	101,252
[40–49]	54,747	57,949	58,791	59,257	191,059	202,614	207,224	214,524	205,365	213,349	208,174	205,690
[50–59]	64,775	69,473	71,299	72,917	237,559	251,211	258,342	267,456	277,900	287,245	279,901	275,419
[60–69]	63,728	68,926	71,336	73,042	246,970	263,974	275,104	286,604	321,406	333,187	326,665	321,077
[70–79]	65,931	70,339	73,267	75,663	220,607	237,516	251,242	265,031	302,742	311,900	309,532	308,738
80+	87,211	96,849	104,537	110,790	163,661	183,176	198,146	213,288	234,114	247,833	250,120	251,993

**Table 4:** Total expenditure (kEur) and governmental co-payment (kEur) with psychotropic drugs.

	Antipsychotics				Antidepressants				Benzodiazepines			
	2016	2017	2018	2019	2016	2017	2018	2019	2016	2017	2018	2019
Total expenditure (1000 Eur)												
All	62,828	71,128	72,268	74,845	66,464	77,535	81,878	88,827	32,878	36,868	36,859	36,873
Age												
[18–29]	6507	7388	7809	8132	1907	2255	2431	2800	606	663	677	711
[30–39]	9662	10,654	10,439	10,367	4613	5037	5000	5219	1685	1760	1682	1641
[40–49]	12,630	14,367	14,718	15,291	9709	11,170	11,625	12,471	3952	4343	4296	4294
[50–59]	11,990	13,446	13,517	14,087	13,319	15,258	15,878	17,021	6149	6832	6769	6722
[60–69]	8958	10,257	10,461	10,963	14,840	17,198	18,147	19,545	7677	8603	8569	8501
[70–79]	6599	7528	7742	7974	13,270	15,639	16,724	18,373	7375	8298	8338	8373
80+	6482	7487	7583	8031	8806	10,979	12,074	13,398	5435	6370	6529	6631
Region												
Alentejo	5081	5850	5949	6136	5181	5988	6325	6782	2377	2618	2573	2544
Algarve	2390	2728	3077	3330	2084	2393	2525	2741	1092	1244	1281	1321
Centro	16,404	18,377	18,915	19,631	17,164	19,477	20,343	21,859	8447	9386	9409	9371
Lisboa	19,496	22,658	23,331	23,864	17,870	21,301	22,475	24,337	7526	8587	8548	8573
Norte	19,457	21,515	20,996	21,884	24,165	28,376	30,211	33,108	13,436	15,034	15,048	15,065
Government co-pay (1000 Eur)												
All	54,925	59,728	60,414	63,290	23,908	28,254	29,810	32,355	14,671	16,040	15,771	15,655
Age												
[18–29]	5684	6369	6712	7032	550	639	687	790	230	248	247	258
[30–39]	8523	9257	9066	9043	1367	1493	1483	1551	656	675	634	615
[40–49]	11,117	12,408	12,679	13,283	3001	3445	3588	3850	1560	1678	1627	1618
[50–59]	10,535	11,474	11,516	12,134	4368	5009	5208	5598	2475	2696	2624	2590
[60–69]	7787	8498	8648	9211	5363	6280	6605	7122	3375	3698	3609	3549
[70–79]	5699	5986	6110	6424	5429	6493	6896	7532	3614	3925	3868	3831
80+	5580	5737	5684	6164	3830	4895	5344	5912	2761	3119	3162	3194
Region												
Alentejo	4465	4915	4989	5218	1983	2325	2461	2658	1097	1180	1140	1112
Algarve	2089	2305	2597	2850	710	818	863	946	463	513	518	530
Centro	14,330	15,437	15,883	16,691	6345	7402	7730	8295	3848	4226	4160	4124
Lisboa	16,985	19,098	19,564	20,211	6046	7281	7709	8387	3172	3509	3429	3396
Norte	17,056	17,974	17,381	18,320	8825	10,428	11,047	12,069	6090	6612	6525	6492

### Prescription and consumption profile by class of psychotropic drugs (2016–2019)

Tables 1 and 2 (and corresponding trend visualization in supplementary Figure A1) disclose the consumption rates (DIDs), number of patients, and expenditure volumes during the period of analysis for the three classes of psychotropic drugs. Significant growth is observed in the number of patients with prescribed antidepressants and antipsychotics (Fig. A1a), representing a 20% increase between 2016 and 2019 in both classes—approximately 250,000 new patients with prescribed antidepressants and 100,000 new patients with prescribed antipsychotics. The number of patients with prescribed benzodiazepines, although stable, is considerably high, 1.5 M (15% of the Portuguese population).

**Figure 1:**
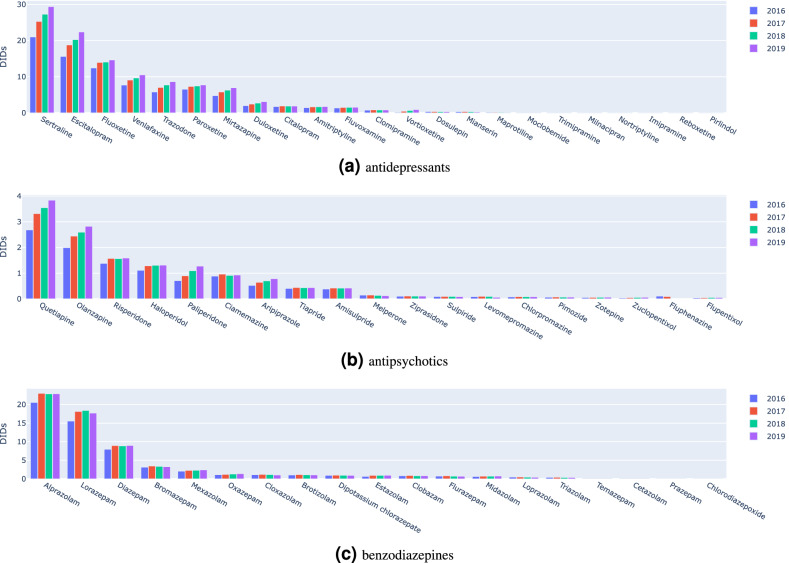
Defined daily dose per 1000 inhabitants-days (DIDs) per psychotropic drug (2016–2019).

The progression of DID consumption rates per active ingredient is provided in Fig. 1 (patient volume progression provided in supplementary Fig. A2). When considering antidepressant prescriptions, we find an increasing trend for selective serotonin reuptake inhibitors where sertraline and escitalopram are among the top 3 most prescribed AD as well as those with alpha-2 antagonistic action, particularly trazodone and mirtazapine. The prescription of serotonin and noradrenaline reuptake inhibitors (SNRIs), such as venlafaxine and duloxetine also shows an upward trend in contrast to tricyclic antidepressants showing a clear downward trend. Except for amitriptyline, the prescription of dosulepin, maprotiline, trimipramine, nortriptyline, imipramine and pirlindole seems to suggest discontinuation of their use.

Considering antipsychotics, there is a clear prescription tendency towards atypical antipsychotics, with a particular incidence on quetiapine (with an increase of approximately 20,000 patients per year), risperidone and olanzapine. Among the typical antipsychotics, amisulpride stands out with a stable prescription rate throughout the study period. The consumption rates of zuclopenthixol, ziprasidone, pimozide, flupentixol and fluphenazine are increasingly residual.

The prescription of benzodiazepines appeared stable along the target years, with a preference for the prescription of alprazolam, diazepam, lorazepam and bromazepam, and an increasing prescription trend of mexazolam.

### Demographic profiles

Consider the gender and age distribution of the consumption rates and patient volume in Tables 1 and 3 (and supplementary visuals along Figures A3 to A8). We observe a higher incidence of psychotropic drug consumption and prescription in women in all our sub-samples, and significantly higher consumption rates (DID) in the age groups above 40 years (*p* value < 0.001, quarter estimates). Those at a younger age seem to represent a considerably smaller share of prescription and yet this group appears to be growing expressively (Tables 1, 3; 18–29 years). Finally, we observed that the volume of expenditures is on par with the incidence of prescriptions in the female population (Fig. A3). However, in the male population, younger patients seem to represent a larger spending per volume of prescriptions (Fig. A3). The proportion of antidepressants, antipsychotics and benzodiazepines does not differ significantly by age group. However, there is a predominance of antidepressant use over benzodiazepines in the younger age groups. Considering the gender distribution per active ingredient (Figs. A7, A8), we observe that most drugs do not generally show a significant deviation from gender distribution expectations (*p* value < 0.001) when considering normalization to the magnitude of each gender group, with few exceptions, including tiapride which is more frequent in men.

### Medical specialty of the prescriber

**Figure 2:**
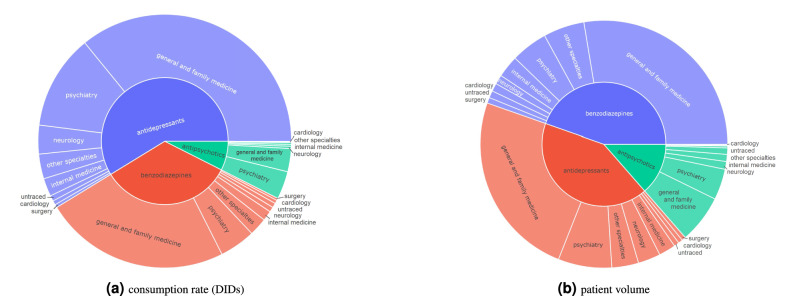
Prescribing medical specialty, 2018–2019.

An initial view of the consumption rate and patient volume by medical specialty and class of psychotropic drugs is presented in Fig. 2 (complementary results provided in supplementary Figures A12 and A13). First, we observed that over two-thirds of benzodiazepine prescriptions were carried out by family physicians, with psychiatrists prescribing only 11% and internal medicine five percent. Secondly, and analogously, there is similar representativeness in the prescription of antidepressants, with Family Practice comprising more than 60% of all prescriptions. In this class, psychiatry and neurology specialties have a higher share of prescriptions, representing approximately 20% and 6% of the total volume of prescriptions, respectively. Third, Family Practice was also the specialty that most prescribed antipsychotics (47% of prescriptions), followed by psychiatry, which together were responsible for approximately 80% of the antipsychotic prescriptions. Considering the proportion of each psychotropic drug class prescribed by medical specialty (Figure A13), benzodiazepines are the most prescribed, while antipsychotics represent less than 10% of total prescriptions in more than 80% of the specialties. Alongside, we observe considerable variations in the prescription incidence per class of psychotropic drugs between medical specialties.

**Figure 3:**
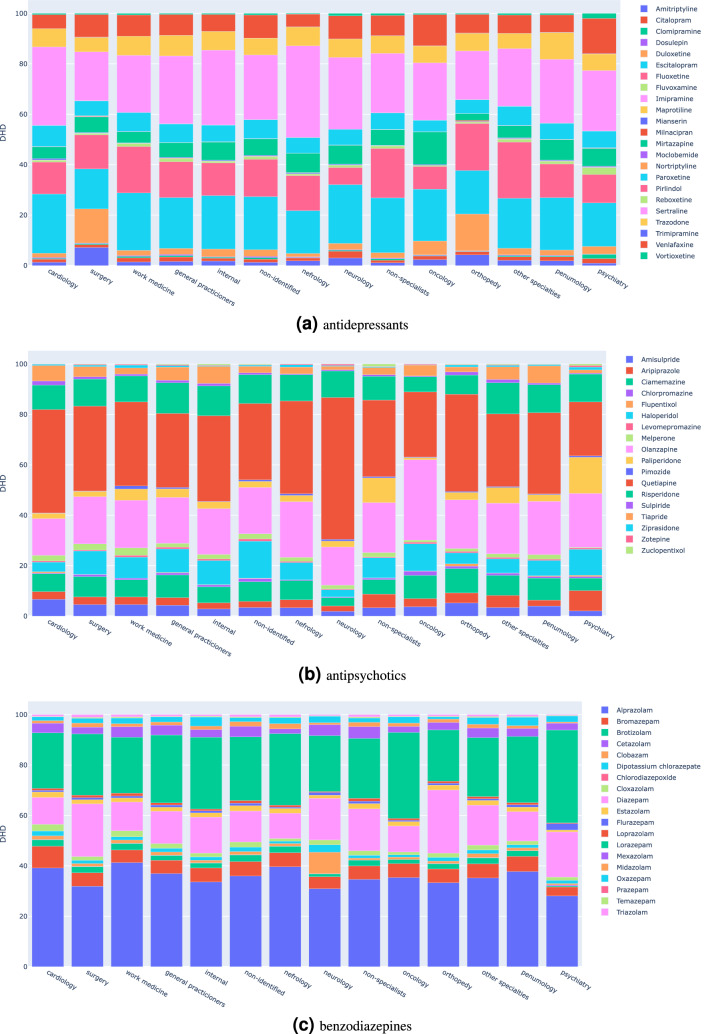
Distribution of psychotropic drugs (DIDs) prescribed per medical specialty, 2018–2019.

Looking in detail at the distribution of active ingredients per class in Fig. 3, we can observe that the proportion of the different antidepressants does not significantly differ among specialties. Nevertheless, we observe that surgery, neurology, orthopaedics and rheumatology prescribe more amitriptyline and duloxetine than the remaining specialties. In turn, psychiatrists prescribe some active substances with lower representation in other specialties, including citalopram, clomipramine, fluvoxamine and vortioxetine.

In the context of antipsychotic prescriptions, there is also a balanced drug distribution per specialty, with a clear cross-sectional preference for quetiapine. The use of haloperidol in oncology exceeds other antipsychotics in this medical specialty, differently from what happens in all other specialties. This observation could be motivated by off-label uses (e.g., inhibiting nausea) rather than treatment of psychosis. Similarly to what happened with antidepressant prescriptions, we observed that psychiatrists prescribed more assorted antipsychotics (e.g., aripiprazole and paliperidone), almost absent from the prescription profiles of other specialties.

Finally, in terms of benzodiazepines, we also observed a consistent distribution of drugs by specialty. Specialties with lower benzodiazepine prescriptions tend to prefer diazepam, while specialties with more active prescription profiles tend to prefer alprazolam. In psychiatry and oncology, the preference for lorazepam also stands out.

### Geographical distribution

**Figure 4:**
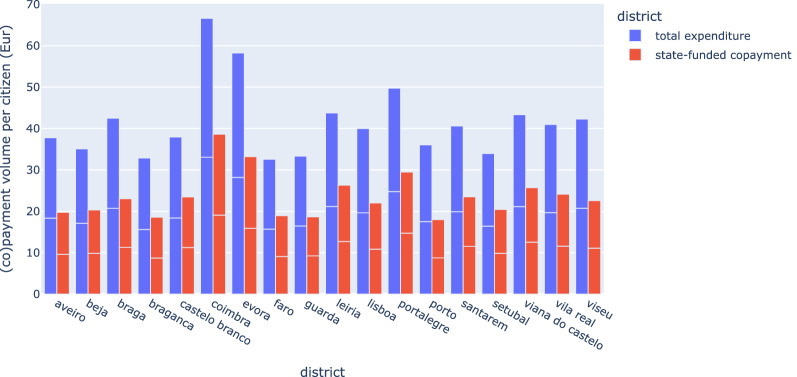
Annual expenditure per district, normalized by citizen, 2018–2019.

Tables 1, 2, 3 and 4 decompose consumption rates, patient volume, and total-and-relative expenditures per geography (visualization in supplementary Figures A9 and A10). Patient incidence per geography is standardized by the age distribution of citizens per region, and region allocation is determined based on primary health care activity center. The geographical distribution of consumption rates (Table 1, Figure A9) reveals some discrepancies. The Algarve, for example, is the region with the lowest consumption of antidepressants and anxiolytics, and the North of Portugal registered at the same time the lowest consumption of antipsychotics and highest of anxiolytics. Figure 4 breaks down the expenditure analysis by district to acquire a finer spatial granularity with the aim of identifying areas with deviating levels of (total and copay) expenditure per citizen in the Portuguese territory. Districts with an incidence of prescription per resident above the average include Coimbra, évora and Portalegre while Faro and Setúbal have incidences below the average. The analysis of the variability between quarters confirms the statistical significance of the differences reported (*t*-test, $\alpha$ = 0.001, quarter estimates). The Center of Portugal region stands out as the one with the highest expenditure per user, both absolute and only considering state co-payments. Porto and Guarda districts, despite presenting a number of patients in line with the average, register an expense resident below average due to a lower number of prescriptions per user.

## Discussion

Our data suggest an overall increase in the prescription rates, the volume of expenditure, the number of prescriptions, and the number of patients prescribed with psychotropic drugs over the years. The Portuguese trend is similar to other worldwide trends^3–7^. This increase was greater between 2016 and 2017, probably reflecting a change in policy regarding the mandatory use of PEM, thus increasing the prescription registry. Among the three drug classes, antidepressant use registered largest increase during this period, with more 45.13 DID than the ones prescribed in 2016, corresponding to an increase of 47%. The last studies on the matter in Portugal reported a 20% increase per year in the antidepressant consumption rates between 2000 and 2012^8^. This new data shows that this trend seems to be slowing down, yet solidly positive. It is also important to notice that, especially within this class, this trend may have accelerated after 2019 considering the impacts of the COVID-19 pandemic.

When considering the OCED report on antidepressant consumption, our results are in line with estimates up to 2017^13^, confirming that Portugal is significantly above the OECD average antidepressant use (63.3 DID). This continuous increase may result from an improved recognition of mental disorders and accessibility to treatment, including better clinical guidelines. However, it is also likely to be associated with increased societal stressors; the worsening of medical intervention, namely shorter consultation length and lesser frequency, the inaccessibility to other forms of interventions such as psychosocial; and difficulties in tapering off these drugs due to withdrawal phenomena or rebound^16,17^.

SSRIs stand out as the most prescribed antidepressants, in line with the guidelines for depressive and anxiety disorders and previous evidence from other countries^18–20^. This type of antidepressant, particularly sertraline, is probably preferred by physicians due to its well-documented great combination of efficacy and acceptability^21^. Trazodone, an atypical antidepressant, is the fifth most prescribed drug in the country, probably due to its use as an add-on during adjustment reactions and depressive episodes in patients with insomnia. In fact, data from the United States of America show that most trazodone prescriptions are explained by its off-label use for the treatment of primary or secondary insomnia^22^. In another stance, tricyclic antidepressants (TCA) seem to have become discontinued, perhaps due to their low safety and tolerability. However, as observed in similar studies^23,24^, amitriptyline stands out in this scenario, probably, again, derived from its Federal Drug Association (FDA) non-approved use for the treatment of insomnia, chronic pain or bladder pain syndrome^25,26^. The use of TCAs for non-psychiatric indications may also be reflected by the relatively higher prescription rates of these drugs by non-psychiatric specialties.

Portugal also registers a clear upward trend in the prescription of antipsychotics, with a 41% increase in this period, especially among atypical antipsychotics. Quetiapine stands out as the most prescribed antipsychotic in the country. This may be partly explained by its off-label use for non-approved FDA indications such as anger management, dementia and insomnia (Office of Public Affairs 2010). Despite the risk of metabolic syndrome associated with their use, risperidone, quetiapine and olanzapine are still prescribed to a greater extent than other antipsychotics, possibly due to their key sedative properties and approval in non-psychotic mood disorders. Amisulpride prescription also stands out perhaps due to its use in depressive symptoms in those with assorted mental symptoms (e.g. conversion/somatic), as an add-on for bipolar type-1 disorders^27,28^, rather than its sole use as an antipsychotic agent^29^. As observed with tricyclic antidepressants, several antipsychotics are not part of Portugal’s prescription choices. Reasons might include unsatisfactory side effect profile (e.g., phenothiazines which have high extrapyramidal effects and low antipsychotic action)^30–34^. In the transition to second-generation antipsychotics, ziprasidone seems an exception. Perhaps this is due to the existence of alternative drugs choices with better safety and tolerability profile (e.g., vs aripiprazole, with lower rates of hyperprolactinemia, sedation, and tardive dyskinesia) and efficacy (e.g., vs olanzapine and risperidone)^35^. The growing use of antipsychotics could also be explained by their increasing use in Bipolar Disorder^36^, and the growing familiarity of its prescription among GPs, able to manage them better than in the past and less afraid to use them^20,30,32^.

The prescription of benzodiazepines, despite the sudden increase in the first year of the study, seems to be stable from 2017 to 2019. This might refer not only to a growing consciousness of their potential hazards (e.g., falls, dependence, and/or cognitive impairment) but to the crescent shift to SSRIs^37,38^. However, it is worth noticing that this overall trend is supported by the decrease in prescriptions among younger adults (18–69 years), while an increase for patients above 70 years old is still evident. This is of particular concern as benzodiazepines are considered a potentially inappropriate medication (PIM) in this population and their prescription should be avoided^39,40^. With respect to the general choice of benzodiazepines, our results go along with previous evidence where alprazolam, diazepam and lorazepam are the most commonly prescribed anxiolytic drugs^37,38^. Indeed, alprazolam was the most prescribed in our sample, likewise prescription practices of most countries in the world^41^. While short half-life benzodiazepines with partial agonism and preference to alpha 2 subunit of GABA receptor, such as alprazolam, may represent fewer hazards (e.g., disturbances of consciousness, cognitive impairment, risk of fall)^42^ they are more frequently associated with dependence^43^. Alprazolam use is seconded by diazepam, a long-acting benzodiazepine with more than 50 years of history, while lorazepam takes up the 3rd place in the prescription profile which is along with its extensive and safe use in situations where there is liver disease or damage^44^.

### Demographic and geographic correlates

Although the use of psychotropic drugs by younger groups represents a smaller share, they seem to grow expressively from 2016 to 2019, in line with what’s happening in other countries^45^. This can either moved by societal stressors, earlier diagnosis, and/or failure to provide other forms of early intervention (e.g. psychotherapy). This increase is in conformity with previous evidence that considers aggravated societal factors (employment insecurity, low income, reduced social benefits and recession) impacting the mental health in youth^46^. The prescription of psychotropics in subjects over 50 years constitutes more than 50% of the Portuguese total share and, while this can be driven by the ageing of the Portuguese population^47^, it can be aggravated by the cumulative effect of the use of psychotropic drugs in adaptive reactions and kept beyond their actual needs. In all three drug classes, the consumption rates increased with age. We observed a linear rise among antidepressant and benzodiazepine consumption, with an increase of 49,5 DID of antidepressants and 28.4 DID of benzodiazepines per age group. In the case of antipsychotic use, there is a slow increase from 18 to 49 years which becomes stable until reaching the group above 80 years old, where it suddenly increases by 63.8%, a fact that could be explained by their use in dementia syndromes, amongst other conditions^1,48^. While the higher incidence of antipsychotics in the > 80 years old group might correspond to actual needs for behaviour control and other neuropsychiatric symptoms^49^, there is evidence alerting that, in some contexts, only 10% of psychotropic drugs in the elderly are correctly prescribed^50^. Studies and policies to treat mental disorders in elderly ages are thus fundamental to assess the current status and the role of non-pharmacological intervention on issues such as loneliness that could reduce the use of psychotropic drugs. Considering benzodiazepines, an increase has been observed for young populations in many countries, frequently associated with long-term use patterns against international and national guidelines^51^. In contrast, in the Portuguese case, a higher proportion of this age group uses antidepressants. While we hypothesize that the clinicians are aware of the paradoxical effects of benzodiazepines in different ages^52^ (e.g., adolescents and individuals with +65 years), including cautions related with the risk of addiction, these results can be complementarily driven by the increasing evidence of antidepressants as the long-term choice for the treatment of anxiety^53^ and depressive disorders^54^.

Women represent the group with the highest consumption rates (DIDs) in general, with approximately three times more antidepressants, as well as two times more benzodiazepines, than men. The observed gender distribution of psychotropic drug use is supported by previous Portuguese^46^ and worldwide^55^ studies. Discussion on this topic includes the possibility that men could be under-treated and women over-treated with antidepressants^56^. While some consider adaptive reactions to be more common in women, the most frequently considered reason for this clear discrepancy is that internalized stigma for help-seeking might hinder the medicalization of suffering in males. Only in the antipsychotic prescriptions do men register higher values with a 14% higher consumption rate. Tiapride use in Alcohol Use Disorders, more prevalent in men^57^, might explain this fact.

The geographical distribution of prescriptions (adjusted for age) also reveal important discrepancies. The North region records the highest prescription rates of antidepressants and benzodiazepines, but the lowest for antipsychotics. Our results are against recent evidence suggesting heavy use of antipsychotics in rural areas in the north of Portugal^58^. The Algarve region, on the contrary, always assumes low rates in comparison to other regions. This may reflect a chronic lack of access to primary care in this region, with some patients being followed up in central hospitals outside Algarve, as well as an increased dependency of the private sector. We also hypothesize that the exposure to blue surfaces (i.e. the sea) and a warm and dry climate, both factors associated with better mental health outcomes, can also play a role. Several previous studies suggest that socioeconomic status (inc. health insurance) can underlie geographical differences in prescription patterns^59^. Future studies are necessary to outline the determinants for higher prescription in each region and support the implementation of subsequent mitigation strategies.

### Medical specialties

As seen in Fig. 2 on the medical speciality responsible for the prescription, General Practitioners (GPs) preside over all other specialties in all three-drug classes, while psychiatry and neurological specialty are together responsible for only 21% of overall prescriptions. In Portugal, primary care services are a strong component of the National Health Service, with GPs being responsible for the management of non-complex affective disorders, as well as the follow-up of chronic and stable psychiatric disorders. This is along with evidence in other countries where GPs are responsible for most drug prescriptions^17,60,61^. This observation outlines the need for intervention in the training and clinical decision support on the treatment of mental disorders and precision psychopharmacology among GPs, and the introduction of good practice goals in the annual action plans of the primary care units. Our results for antidepressants show a higher use of citalopram, fluoxetine and sertraline as observed in previous studies^17^ and there seems to be evidence of off-label prescription^62^. GPs seldomly use recent psychotropic drugs^61^ or else rely on direct marketing strategies to identify new treatments^63^ which are adopted under the premise that these new drugs are more effective^61^. Although the use of benzodiazepines and antidepressants is not restricted to the treatment of mental disorders, our figures also raise important considerations regarding the monitoring of patients prescribed for mental disorders by other medical specialities, including psychiatry, neurology, and surgery. Short and long term treatment with benzodiazepines might risk being outdated or beyond rationale from the National Institute for Health and Care Excellence (NICE) if not validated by proper specialities (e.g., neurology and psychiatry). Liaison psychiatry should play an important role here. We further observed that surgery, neurology, orthopaedics and rheumatology prescribe significantly more amitriptyline and duloxetine than the remaining specialities, an observation that is associated with the role of these drugs in neuropathic pain control and urinary stress incontinence^64^. The use of haloperidol in Oncology could also be motivated by off-label uses (e.g., inhibiting nausea) rather than treatment of psychosis. The case of antipsychotics use, which the FDA and EMEA approved for symptomatic treatment of psychoses and affective disorders, the extensive prescription rate by GPs could constitute either an off-label use for insomnia or behavioural symptoms, as well as the result of a long-term treatment of stabilized effective schizophrenia spectrum disorders under the care of a GP.

### Expenditures

Considering state-funded expenditure, antipsychotics are the class with highest governmental copayment (> 50% of investments among psychotropic drugs, a 6.33 Eur cost per citizen in 2019), partly driven by the high subsidization of these drugs, increasing share of atypical antipsychotics (with considerably higher pricing than first generation alternatives), and the low impact that the introduction of new generic drugs yields in the existing branded drugs^65^. The Portuguese subsidization strategy for antipsychotics is moderately aligned with other countries^66^, grounded on the impact over accessibility and medication adherence^67^, as well as downstream healthcare cost benefits, particularly those costs pertaining to the end-to-end care provided to those individuals with schizophrenia or bipolar I disorders^66,68,69^. Antipsychotic expenditures with undesirable impacts on healthcare costs, including those expenditures attributed to antipsychotic polypharmacy (low-value care)^70^ or inadequate selection (with regards to the active ingredient and delivery mode)^71^, should be also considered.

In spite of the high benzodiazepine prescription prevalence in Portugal, the lower production costs and adherence to generic dispensation caps the expenditures of this class (< 15% of the state-funds among psychotropic drugs, an approximate 1.50 Eur cost per citizen in 2019). Nevertheless, the critical downstream healthcare and socioeconomic impacts of benzodiazepine malprescription should be noted^72^.

Although antidepressants represent < 30% of state-funded expenditures with psychotropic drugs in 2019, they represent the class with the highest total expenditure, with a +33% expenditure growth (between 2016 and 2019) and an estimated 9 Eur quote per citizen in 2019. This appears to be a direct result of high prescription incidence and growth. Yet, it is in contrast with other European countries where the total expenditures of antipsychotics already overtook those of antidepressants, hypothesized to be driven by the lower incidence of antidepressant prescriptions in these countries, together with an increasing number of prescriptions of some antipsychotics not being restricted to patients with serious mental disorders^73^. Complementary to prevalence, moderate increases in average pricing of some antidepressants can be further accounted as a possible driver^74,75^.

### Limitations

First, our study excludes non-benzodiazepine anxiolytic drugs such as zolpidem, limiting our results to the class of benzodiazepines, a care to be undertaken when establishing comparisons against DID references in other countries and historical estimates in Portugal. Although DDD and DID are still considered to be the reference indicators for pharmacoepidemiology studies alike, they can fail to show us a more detailed relation between prescriptions and the underlying diagnosis. Due to multi-level privacy concerns, several clinical inputs (e.g. diagnosis information) could not be collect at a nationwide level, which would be pivotal to understand the psychiatric dynamics that go along with the depicted trends, thus preventing us from inquirying whether the changes in prescription and consumption are related with the increasing prevalence of mental disorders. This study also fails to consider some other important psychotropic drugs, such as lithium and others with mood-stabilizing properties (carbamazepine and di-valproate). Their future inclusion is important to understand their pharmacoepidemiology and if their prescription is being supplanted by second-generation antipsychotics as evidenced in previous studies. Despite the taken care in tracing the residence of patients by primary healthcare service, low-to-moderate geographical discrepancies may occur due to the centralized management of mental healthcare treatment in Portugal. In addition, we were not provided with complete prescription registry from the autonomous regions of Azores and Madeira due to the non-mandatory use of electronic registry in these regions throughout the period of the cohort study. The inclusion of these regions is expected in the future for an all-encompassing analysis of the country. Finally, there is a remnant use of paper prescriptions up to 2017, nevertheless credited by the Health Ministry as being largely residual and thus not determinant for analysis or discussion.

## Conclusions

This study is the first to comprehensively examine and discuss the prescription patterns of psychotropic drugs in Portugal across 2016–2019. Several of the acquired results are in line with the existing body of research in other countries—an increase in consumption rates and volume of expenditure, as well as a consolidation trend towards the prescription of specific groups of psychotropic drugs. In particular, we observed an increase in the consumption (>3 0%) and expenditure (37M Eur) of antidepressants and antipsychotics between 2016 and 2019, and a stabilization in the use of benzodiazepines with an overall consumption by > 15% of the Portuguese population. With few exceptions, tricyclic antidepressants and typical antipsychotics seem to be under discontinuation suggesting prescribers are favouring SSRIs and atypical drugs. The use of psychotropic drugs seems to be higher in those of older age and women, and disparate among different Portuguese regions (after correction for age). Sociodemographic, geographical and cost correlates are explored, unravelling relevant drivers to assess the status of psychotropic drug prescription. The analysis of the responsible medical specialities reveals that General Medicine is responsible for approximately 64% of prescriptions, and is arguably less assorted in the choices when considering the changing in the preferred psychotropic drugs by psychiatry and neurology.

Unique aspects of the Portuguese case are also noted and discussed, including the ageing of the Portuguese population, the follow-up of mental disorders in GP, and the economic recession.

Our findings ultimately constitute opportunities for public health initiatives, the design of new practice recommendations for psychotropic drug prescription, medical training programs, the strengthening of protocols between psychiatrists and GPs, as well as psychosocial interventions.

We aim to further assess the patterns of psychotropic drug prescription in Portugal by continuing with the collection and processing of PEM data for the subsequent years, estimating the potential impact yield throughout and after the COVID 19 pandemic in the consumption status. In addition, complementary clinical data (e.g., diagnosis and interventions) can be crisscrossed with the available data to understand the underlying rationale for the different prescription patterns, and set tailor made protocols for deprescribing strategies. As our study further suggests the close monitoring of several prescription patterns, either for their possible long-term or off-label use, we further aim to analyze the prescription registries to study mal-prescription patterns, including problems of polimedication.

## Supplementary Information

Supplementary Information.

## Data Availability

Acccess request to PEM data can be directed to *Serviços Partilhados do Ministério da Saúde* (SPSM) (https://www.spms.min-saude.pt/contactos/) in the presence of the research aims and ethical approval.

## References

[1] IHME. Gbd Compare Data Visualization. *Institute for Health Metrics and Evaluation, IHME Website* (2022).

[2] Mental well-being and social support statistics (2019). In *Health in the European Union* (2022). https://stat.link/pcxvjy.

[3] OECD. Oecd Health Statistics 2019 (2020).

[4] Bachhuber MA, Hennessy S, Cunningham CO, Starrels JL (2016). Increasing benzodiazepine prescriptions and overdose mortality in the united states, 1996–2013. Am. J. Public Health.

[5] Murphy Y, Wilson E, Goldner EM, Fischer B (2016). Benzodiazepine use, misuse, and harm at the population level in Canada: A comprehensive narrative review of data and developments since 1995. Clin. Drug Investig..

[6] Xu L (2021). Trends in psychotropic medication prescriptions in urban china from 2013 to 2017: National population-based study. Front. Psychiatry.

[7] Hálfdánarson Ó (2017). International trends in antipsychotic use: A study in 16 countries, 2005–2014. Eur. Neuropsychopharmacol..

[8] Caldas de Almeida, J. M. *et al.* Estudo epidemiológico nacional de saúde mental. *Lisboa. Faculdade de Ciências Médicas, da Universidade Nova de Lisboa* **1** (2013).

[9] Semoun O, Sevilla-Dedieu C (2015). Psychotropic drug consumption among older people enrolled in a French private health insurance plan. Drugs-Real World Outcomes.

[10] Carrasco-Garrido P (2016). Time trend in psychotropic medication use in Spain: A nationwide population-based study. Int. J. Environ. Res. Public Health.

[11] Gardner DM (2014). Competent psychopharmacology. Can. J. Psychiatry.

[12] de Saúde, C. N. Sem mais tempo a perder: Saúde mental em portugal-um desafio para a próxima década (2019).

[13] OECD & EU. Health at a glance: Europe 2018: State of health in the EU cycle (2018).

[14] Furtado, C. *Psicofármacos: Evolução do consumo em portugal continental (2000–2012) [internet]* (Infarmed, Autoridade Nacional do medicamento e produtos de saúde, Lisboa, 2012).

[15] Ronning M (2006). Recommendations for national registers of medicinal products with validated ATC codes and DDD values. Ital. J. Public Health.

[16] Tobin H, Bury G, Cullen W (2020). Mental illness in primary care: A narrative review of patient, GP and population factors that affect prescribing rates. Irish J. Psychol. Med..

[17] Johnson CF, Williams B, MacGillivray SA, Dougall NJ, Maxwell M (2017). ‘Doing the right thing’: Factors influencing GP prescribing of antidepressants and prescribed doses. BMC Fam. Pract..

[18] Bogowicz P (2021). Trends and variation in antidepressant prescribing in English primary care: A retrospective longitudinal study. BJGP Open.

[19] Dai Cao TX (2021). Prescribing trends of antidepressants and psychotropic coprescription for youths in UK primary care, 2000–2018. J. Affect. Disord..

[20] Livingston M (2020). Antidepressant prescribing in England: Patterns and costs. Prim. Care Companion CNS Disord..

[21] Zhou X (2020). Comparative efficacy and acceptability of antidepressants, psychotherapies, and their combination for acute treatment of children and adolescents with depressive disorder: A systematic review and network meta-analysis. Lancet Psychiatry.

[22] Stahl SM (2009). Mechanism of action of trazodone: A multifunctional drug. CNS Spectr..

[23] Lockhart P, Guthrie B (2011). Trends in primary care antidepressant prescribing 1995–2007: A longitudinal population database analysis. Br. J. Gen. Pract..

[24] Saragoussi D, Chollet J, Bineau S, Chalem Y, Milea D (2012). Antidepressant switching patterns in the treatment of major depressive disorder: A general practice research database (gprd) study. Int. J. Clin. Pract..

[25] Sultana J (2014). Changes in the prescribing pattern of antidepressant drugs in elderly patients: An Italian, nationwide, population-based study. Eur. J. Clin. Pharmacol..

[26] Verhaak PF, de Beurs D, Spreeuwenberg P (2019). What proportion of initially prescribed antidepressants is still being prescribed chronically after 5 years in general practice? A longitudinal cohort analysis. BMJ Open.

[27] Zangani C (2021). Efficacy of amisulpride for depressive symptoms in individuals with mental disorders: A systematic review and meta-analysis. Hum. Psychopharmacol. Clin. Exp..

[28] Carta MG, Zairo F, Mellino G, Hardoy MC, Vieta E (2006). An open label follow-up study on amisulpride in the add-on treatment of bipolar i patients. Clin. Pract. Epidemiol. Ment. Health.

[29] Nuss P, Hummer M, Tessier C (2007). The use of amisulpride in the treatment of acute psychosis. Ther. Clin. Risk Manage..

[30] Buhagiar K, Ghafouri M, Dey M (2020). Oral antipsychotic prescribing and association with neighbourhood-level socioeconomic status: Analysis of time trend of routine primary care data in england, 2011–2016. Soc. Psychiatry Psychiatr. Epidemiol..

[31] Marston L, Nazareth I, Petersen I, Walters K, Osborn DP (2014). Prescribing of antipsychotics in UK primary care: A cohort study. BMJ Open.

[32] Morrens M, Destoop M, Cleymans S, Van der Spek S, Dom G (2015). Evolution of first-generation and second-generation antipsychotic prescribing patterns in Belgium between 1997 and 2012: A population-based study. J. Psychiatr. Pract..

[33] Prah P, Petersen I, Nazareth I, Walters K, Osborn D (2012). National changes in oral antipsychotic treatment for people with schizophrenia in primary care between 1998 and 2007 in the united kingdom. Pharmacoepidemiol. Drug Saf..

[34] Shah S, Carey I, Harris T, Dewilde S, Cook D (2011). Antipsychotic prescribing to older people living in care homes and the community in England and Wales. Int. J. Geriatr. Psychiatry.

[35] Gardner DM (2013). Evidence review and clinical guidance for the use of ziprasidone in Canada. Ann. Gen. Psychiatry.

[36] Hayes J (2011). Prescribing trends in bipolar disorder: Cohort study in the united kingdom thin primary care database 1995–2009. PLoS One.

[37] Rosman S, Le Vaillant M, Pelletier-Fleury N (2011). Gaining insight into benzodiazepine prescribing in general practice in France: A data-based study. BMC Fam. Pract..

[38] Šubelj M, Vidmar G, Švab V (2012). Time trends in prescribing habits of anxiolytics and antidepressants in Slovenian family practices (with emphasis on elderly patients). Coll. Antropol..

[39] Society A. G. (2015). American geriatrics society 2015 updated beers criteria for potentially inappropriate medication use in older adults. J. Am. Geriatr. Soc..

[40] O’Mahony D (2018). Corrigendum: Stopp/start criteria for potentially inappropriate prescribing in older people: Version 2. Age Ageing.

[41] Ait-Daoud N, Hamby A, Sharma S, Blevins D (2018). A review of alprazolam use, misuse, and withdrawal. J. Addict. Med..

[42] Hanlon JT (2002). Use of inappropriate prescription drugs by older people. J. Am. Geriatr. Soc..

[43] de las Cuevas C, Sanz E, de la Fuente J (2003). Benzodiazepines: More “behavioural” addiction than dependence. Psychopharmacology.

[44] Ghiasi, N., Bhansali, R. K. & Marwaha, R. Lorazepam (2021). 30422485

[45] Jack RH (2020). Incidence and prevalence of primary care antidepressant prescribing in children and young people in England, 1998–2017: A population-based cohort study. PLoS Med..

[46] Silva M (2020). How did the use of psychotropic drugs change during the great recession in Portugal? A follow-up to the national mental health survey. BMC Psychiatry.

[47] Ćurković M, Dodig-Ćurković K, Erić A, Kralik K, Pivac N (2016). Psychotropic medications in older adults: A review. Psychiatr. Danubina.

[48] IHME. Gbd compare data visualization. *Institute for Health Metrics Evaluation, IHME Website* (2019).

[49] Liperoti R, Pedone C, Corsonello A (2008). Antipsychotics for the treatment of behavioral and psychological symptoms of dementia (BPSD). Curr. Neuropharmacol..

[50] van der Spek K (2016). Only 10% of the psychotropic drug use for neuropsychiatric symptoms in patients with dementia is fully appropriate. The proper i-study. Int. Psychogeriatr..

[51] Sidorchuk A (2018). Benzodiazepine prescribing for children, adolescents, and young adults from 2006 through 2013: A total population register-linkage study. PLoS Med..

[52] Mancuso CE, Tanzi MG, Gabay M (2004). Paradoxical reactions to benzodiazepines literature: Review and treatment options. Pharmacother. J. Human Pharmacol. Drug Ther..

[53] Gomez AF, Barthel AL, Hofmann SG (2018). Comparing the efficacy of benzodiazepines and serotonergic anti-depressants for adults with generalized anxiety disorder: A meta-analytic review. Expert Opin. Pharmacother..

[54] Kendrick T, Taylor D, Johnson CF (2019). Which first-line antidepressant?. Br. J. Gen. Pract..

[55] Boyd A (2015). Gender differences in psychotropic use across Europe: Results from a large cross-sectional, population-based study. Eur. Psychiatry.

[56] Sundbom LT, Bingefors K, Hedborg K, Isacson D (2017). Are men under-treated and women over-treated with antidepressants? Findings from a cross-sectional survey in Sweden. BJPsych Bull..

[57] Glantz MD (2020). The epidemiology of alcohol use disorders cross-nationally: Findings from the world mental health surveys. Addict. Behav..

[58] Ramos S (2021). Antipsychotic polypharmacy and high doses in a rural Portuguese community mental health service. Rev. Portuguesa Psiquiatr. Saúde Mental.

[59] Dennis JA, Gittner LS, Payne JD, Nugent K (2020). Characteristics of us adults taking prescription antipsychotic medications, national health and nutrition examination survey 2013–2018. BMC Psychiatry.

[60] Mercier A (2015). How do GP practices and patient characteristics influence the prescription of antidepressants? A cross-sectional study. Ann. Gen. Psychiatry.

[61] Svensson SA, Hedenrud TM, Wallerstedt SM (2019). Attitudes and behaviour towards psychotropic drug prescribing in Swedish primary care: A questionnaire study. BMC Fam. Pract..

[62] Rubio-Valera M (2012). Psychotropic prescribing in Catalonia: Results from an epidemiological study. Fam. Pract..

[63] Mercier A, Auger-Aubin I, Lebeau J-P, Royen PV, Peremans L (2011). Understanding the prescription of antidepressants: A qualitative study among French GPS. BMC Fam. Pract..

[64] Reddy S, Patt RB (1994). The benzodiazepines as adjuvant analgesics. J. Pain Symptom Manage..

[65] Van T, Guo J, Mallow P, Shelly D (2020). Pnd116 examine the spending and utilization patterns of second-generation antipsychotics based on medicaid claims database. Value Health.

[66] Wong J, Kurteva S, Motulsky A, Tamblyn R (2022). Association of antidepressant prescription filling with treatment indication and prior prescription filling behaviors and medication experiences. Med. Care.

[67] Hamina A, Tanskanen A, Tiihonen J, Taipale H (2020). Medication use and health care utilization after a cost-sharing increase in schizophrenia: A nationwide analysis. Med. Care.

[68] Van Der Lee AP, De Haan L, Beekman AT (2019). Rising co-payments coincide with unwanted effects on continuity of healthcare for patients with schizophrenia in the netherlands. PLoS One.

[69] Broder MS (2019). Atypical antipsychotic adherence is associated with lower inpatient utilization and cost in bipolar i disorder. J. Med. Econ..

[70] Nili M, Iloabuchi C, Sambamoorthi U (2020). Pdg45 low-value care: The association of antipsychotic polypharmacy use to economic burden in non-institutionalized civilian population in the united states. Value Health.

[71] Fu A, Pesa J, Lakey S, Benson C (2021). Pmh13 healthcare resource utilization and costs before and after long-acting injectable antipsychotic (LAI) use in commercially insured young adults with schizophrenia. Value Health.

[72] Malakouti SK (2021). A systematic review of potentially inappropriate medications use and related costs among the elderly. Value Health Region. Issues.

[73] Ilyas S, Moncrieff J (2012). Trends in prescriptions and costs of drugs for mental disorders in England, 1998–2010. Br. J. Psychiatry.

[74] Gomez-Lumbreras A (2019). Study of antidepressant use in 5 European settings. Could economic, sociodemographic and cultural determinants be related to their use?. J. Affect. Disord..

[75] Chen Y (2008). Utilization, price, and spending trends for antidepressants in the us medicaid program. Res. Soc. Adm. Pharm..

