# Supplementary material for: Prepandemic psychotropic drug status in Portugal: a nationwide pharmacoepidemiological profile﻿
Source: Sci Rep. 2023 Apr 27;13:6912. doi: 10.1038/s41598-023-33765-0 (PMC10139661; doi:10.1038/s41598-023-33765-0)
Supplement: Supplementary file 1 — Supplementary Information. [file 41598_2023_33765_MOESM1_ESM.pdf]

Supplementary Material

Prepandemic psychotropic drug status in Portugal: a nationwide pharmacoepidemiological profile

L. Madeira, G. Queiroz, R. Henriques*
* Correspondence: rmch@tecnico.ulisboa.pt

A1 Complementary results

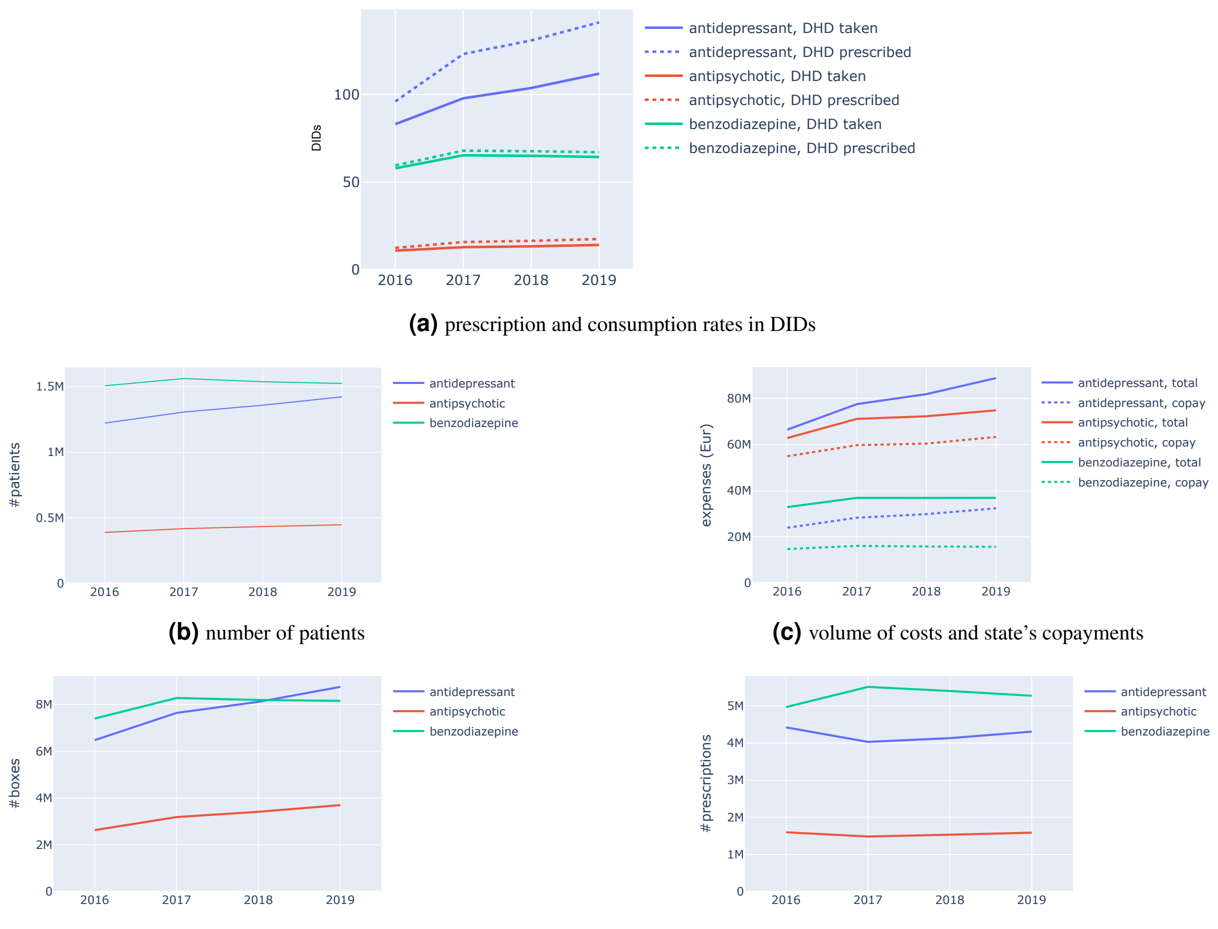

(a) prescription and consumption rates in DIDs

(b) number of patients

(c) volume of costs and state's copayments

(d) number of prescribed and taken boxes

(e) number of prescriptions

Figure A1. Yearly progression of prescription indicators per psychotropic drug class (2016–2019).

Table A1. Summary of the psychotropic drug prescription status in Portugal: prescription rates across demographic variables expressed in Defined Daily Dose per 1000 inhabitants-days (DIDs).

			antipsychotics				antidepressants				benzodiazepines			
			2016	2017	2018	2019	2016	2017	2018	2019	2016	2017	2018	2019
prescription rates (DIDs)	all		12.48	15.82	16.51	17.56	95.97	123.04	130.76	141.09	59.53	67.91	67.62	67.04
	gender	F	11.72	15.00	15.75	16.70	140.53	179.20	189.68	203.91	79.55	90.54	89.77	88.72
		M	13.31	16.72	17.35	18.51	46.77	61.04	65.72	71.74	37.44	42.94	43.18	43.10
	age	[18-29]	5.83	7.41	7.93	8.64	20.15	26.41	28.71	32.45	6.44	7.18	7.06	7.08
		[30-39]	10.76	12.95	12.78	13.13	49.07	59.43	59.72	61.86	21.66	23.01	21.72	20.65
		[40-49]	16.78	20.76	21.17	22.19	95.37	120.43	125.75	133.44	50.77	56.73	55.60	54.47
		[50-59]	19.18	24.23	24.98	26.65	139.25	175.51	182.68	194.32	84.53	95.98	94.97	93.33
		[60-69]	17.22	22.08	23.31	25.00	178.54	226.32	240.07	257.09	117.79	134.76	134.06	132.16
		[70-79]	16.45	21.23	22.84	24.32	211.30	274.11	295.33	323.00	144.39	164.93	165.40	165.34
		[+80]	24.72	33.47	36.72	39.84	221.07	303.36	339.29	378.73	166.80	198.09	202.74	206.02
	region	alentejo	12.71	16.65	17.93	18.89	93.38	117.53	125.81	133.91	47.02	52.64	51.38	50.60
		algarve	11.61	14.72	15.92	17.61	59.59	80.10	86.14	94.09	36.32	42.57	43.37	44.57
		centro	13.85	17.10	17.75	18.77	102.25	126.78	133.28	142.47	64.46	72.81	72.61	71.41
		lisboa	13.32	17.52	18.37	19.35	92.29	123.25	132.01	142.69	44.15	51.35	50.87	50.72
		norte	11.10	13.77	14.23	15.30	99.53	126.78	134.73	146.51	73.86	84.11	84.09	83.46

Table A2. Volume and share of prescriptions per medical specialty and psychotropic drug class (2018–2019).

	**all**		**antipsychotics**		**antidepressants**		**benzodiazepines**	
speciality	volume	share	volume	share	volume	share	volume	share
cardiology	231977	1.0%	11848	0.4%	69892	0.8%	149694	1.4%
general and family medicine	14089362	63.2%	1479899	47.4%	5083039	60.2%	7483668	70.1%
internal medicine	998272	4.5%	168474	5.4%	350966	4.2%	474543	4.5%
medical oncology	90876	0.4%	7472	0.2%	31451	0.4%	51744	0.5%
nephrology	63683	0.3%	5520	0.2%	20006	0.2%	38085	0.4%
neurology	832563	3.7%	160326	5.1%	469398	5.6%	202146	1.9%
non-specialists	168933	0.8%	25117	0.8%	56172	0.7%	86677	0.8%
occupational medicine	114671	0.5%	11885	0.4%	40304	0.5%	62053	0.6%
orthopedics/rheumatology	151611	0.7%	6610	0.2%	59932	0.7%	84736	0.8%
pneumology	95056	0.4%	9254	0.3%	32411	0.4%	53154	0.5%
psychiatry	3966556	17.8%	1055735	33.8%	1719096	20.4%	1188595	11.1%
surgery	211929	1.0%	20261	0.6%	81027	1.0%	109120	1.0%
other specialties	856131	3.8%	86177	2.8%	282776	3.4%	471762	4.4%
non specified	430540	1.9%	71625	2.3%	140771	1.7%	215403	2.0%

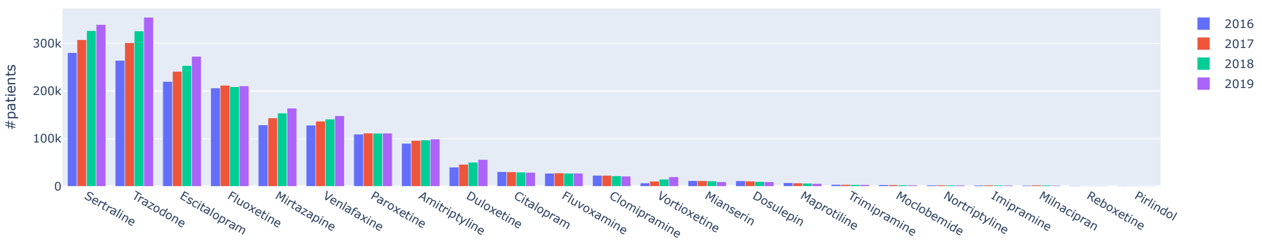

(a) antidepressants

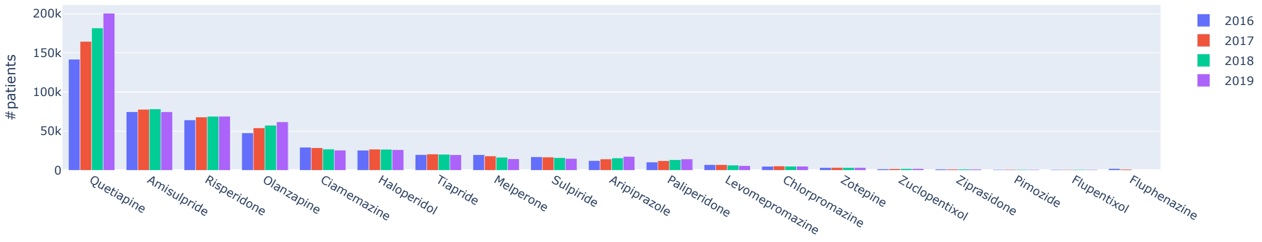

(b) antipsychotics

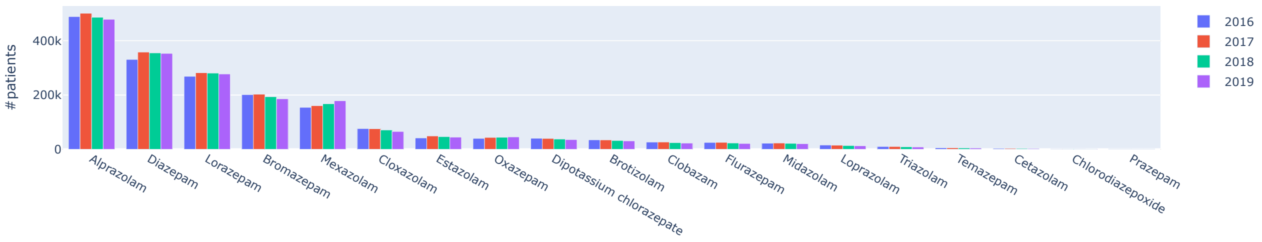

(c) benzodiazepines

Figure A2. Number of prescribed patients per psychotropic drug between 2016 and 2019.

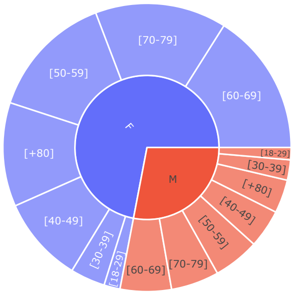

(a) prescription rate (DIDs)

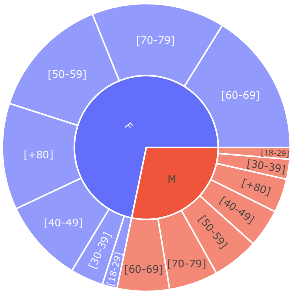

(b) consumption rate (DIDs)

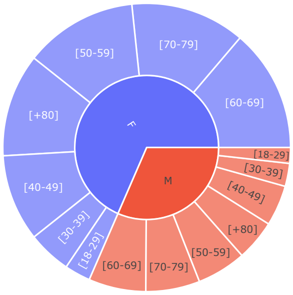

(c) volume of patients

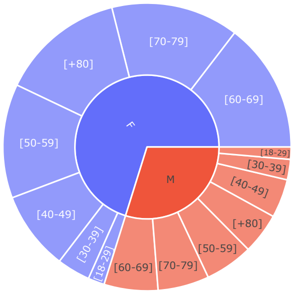

(d) volume of prescriptions

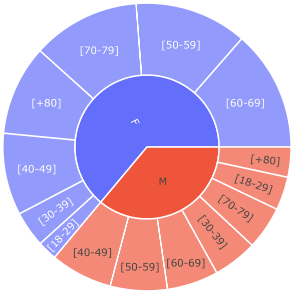

(e) total expenditure

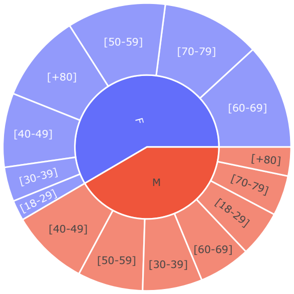

(f) state copayments

Figure A3. Distribution of psychotropic drug prescription volume per gender and age.

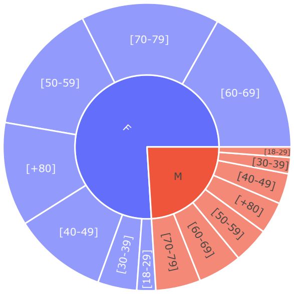

(a) antidepressants

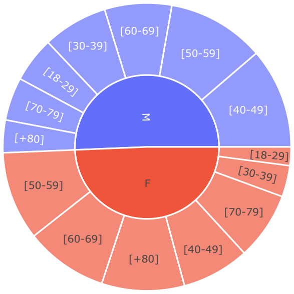

(b) antipsychotics

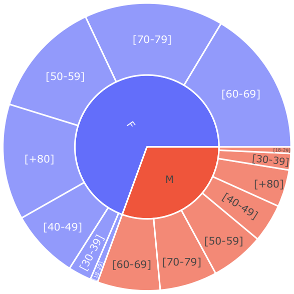

(c) benzodiazepines

Figure A4. Distribution of consumption rates (DIDs) per drug class per gender and age.

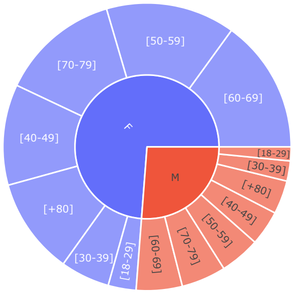

(a) antidepressants

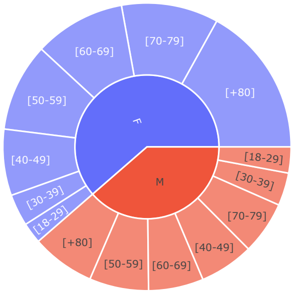

(b) antipsychotics

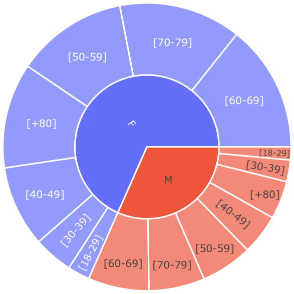

(c) benzodiazepines

Figure A5. Distribution of the number of patients per drug class, gender, and age.

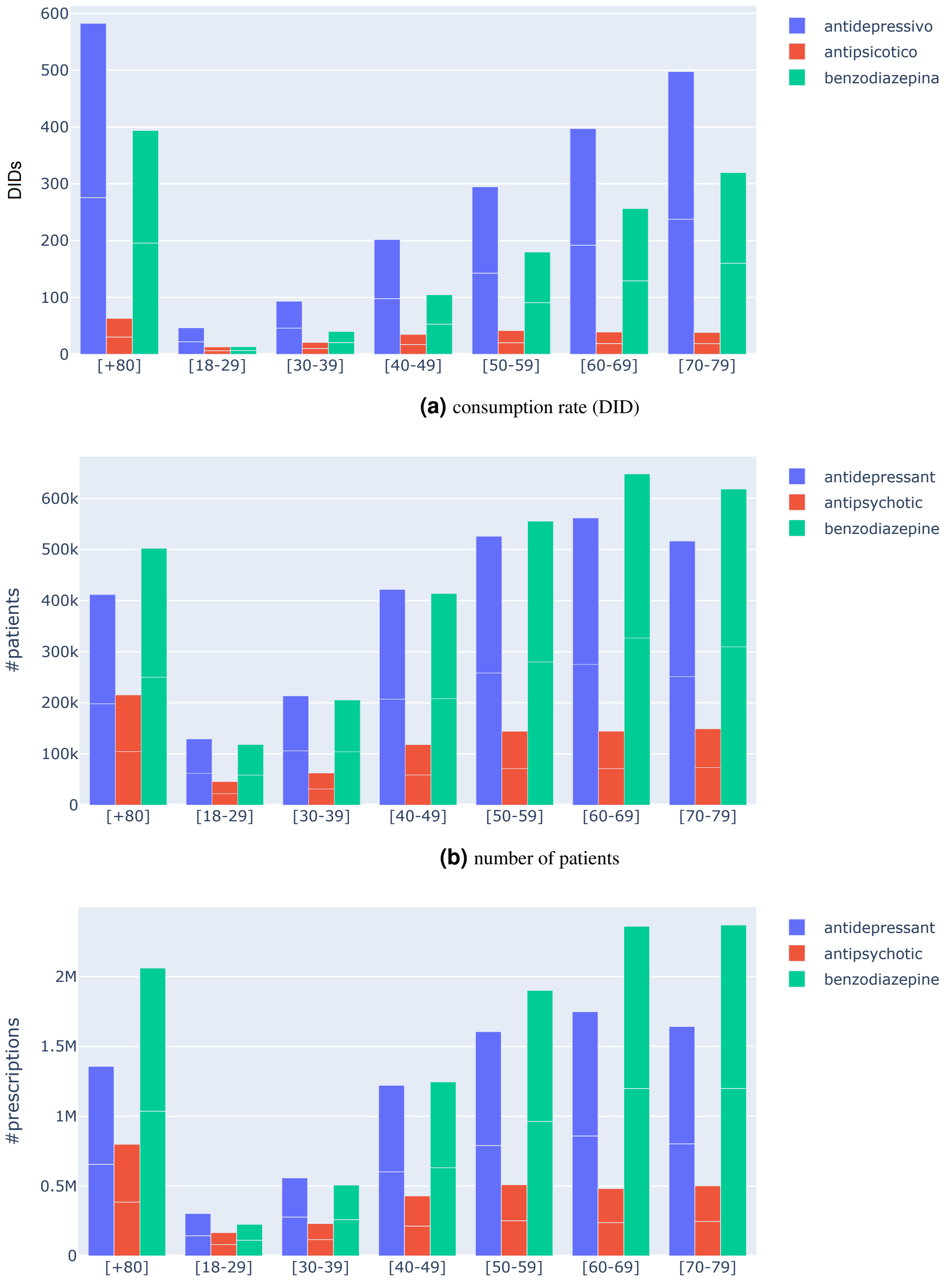

(a) consumption rate (DID)

(b) number of patients

(c) prescription rate (DID)

Figure A6. Distribution of psychotropic drugs per age, 2018–2019.

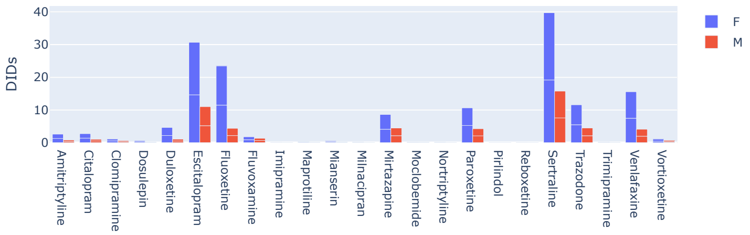

(a) antidepressants

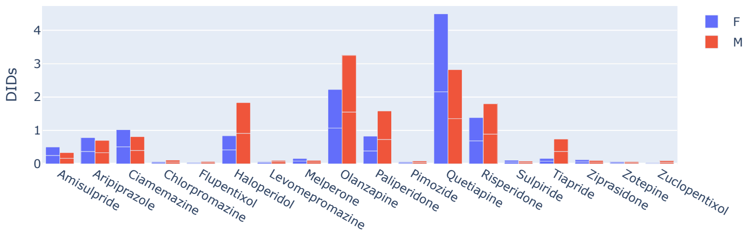

(b) antipsychotics

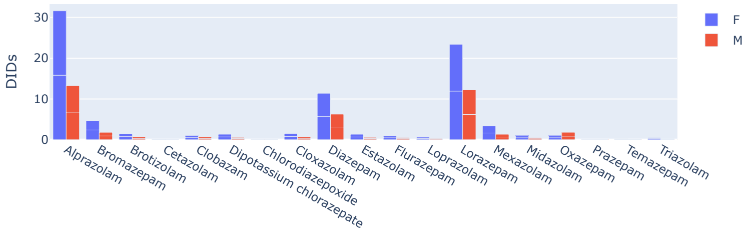

(c) benzodiazepines

Figure A7. Consumption rate in DIDs per drug and gender, 2018–2019.

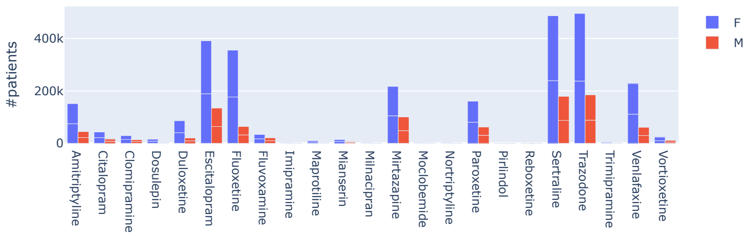

(a) antidepressants

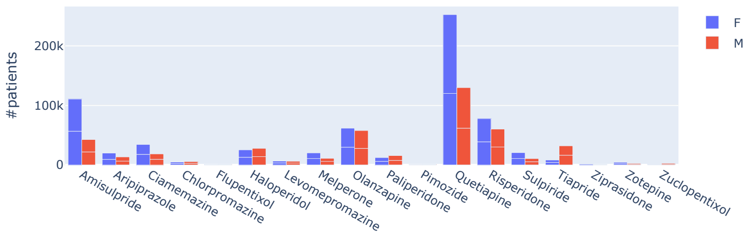

(b) antipsychotics

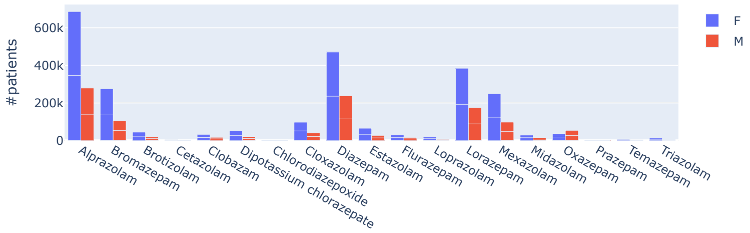

(c) benzodiazepines

Figure A8. Number of patients per psychotropic drug and gender, 2018–2019.

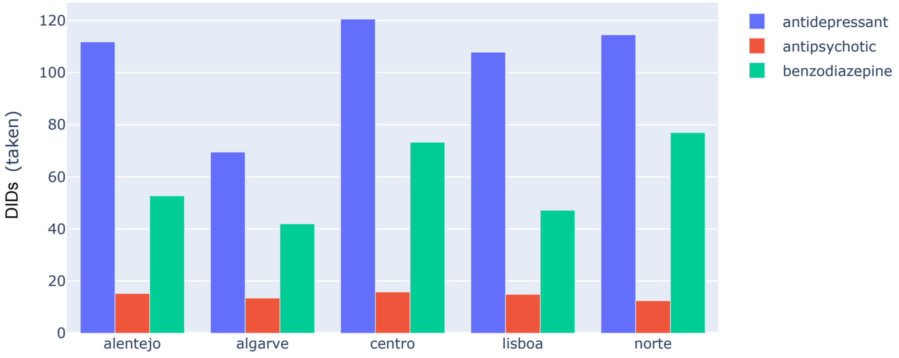

(a) consumption rate (DIDs)

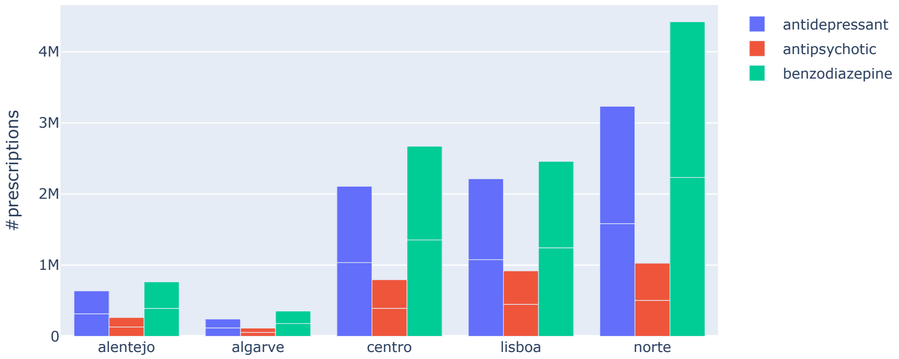

(b) volume of prescriptions

Figure A9. Drug prescription profile per geography after standardization for age groups against census data, 2018–2019.

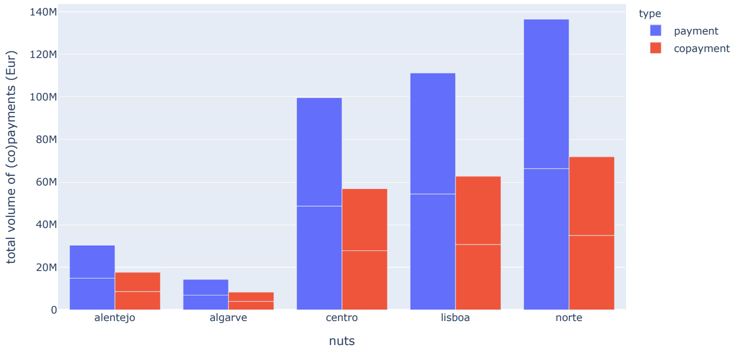

(a) total expenditure in Eur

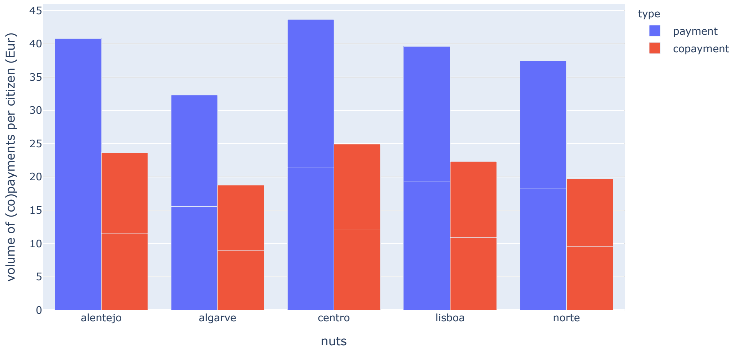

(b) normalized expenditure per citizen

Figure A10. Annual expenditure per geography, 2018–2019.

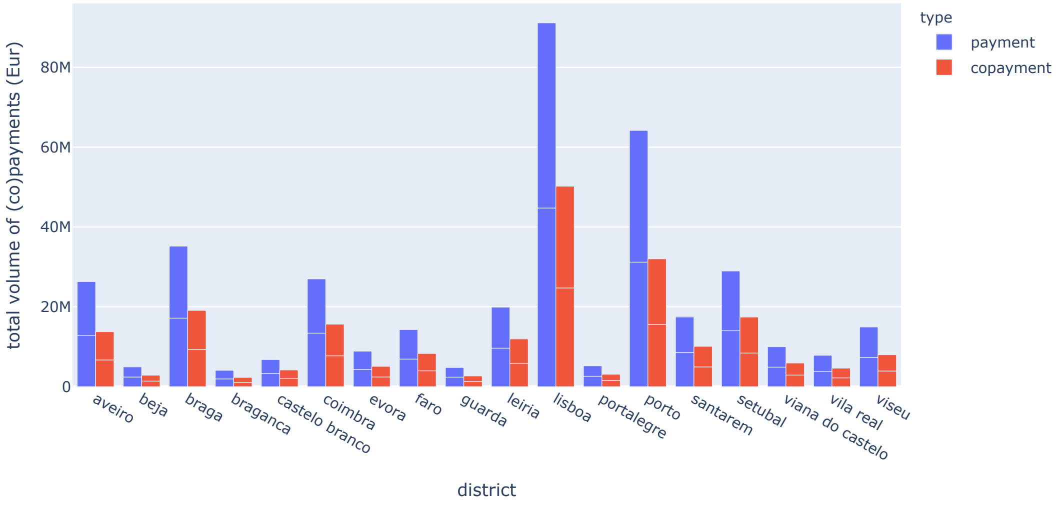

Figure A11. Total annual expenditure per district, 2018–2019.

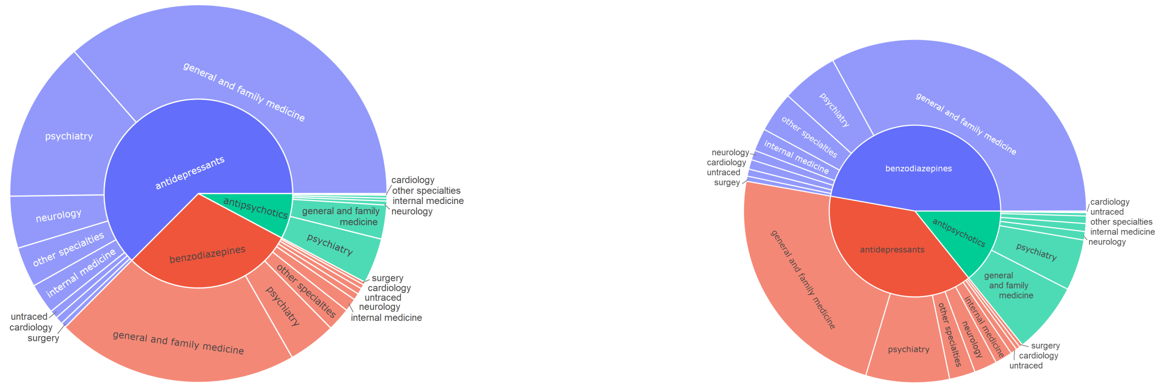

(a) prescription rate (DIDs)

(b) volume of prescriptions

Figure A12. Prescribing medical specialty, 2018–2019.

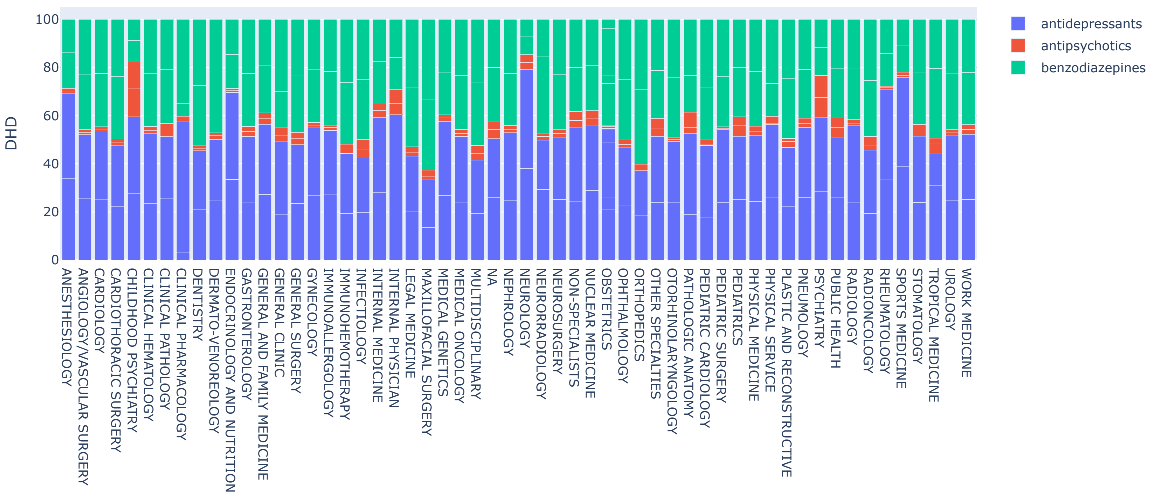

(a) consumption rate (DIDs)

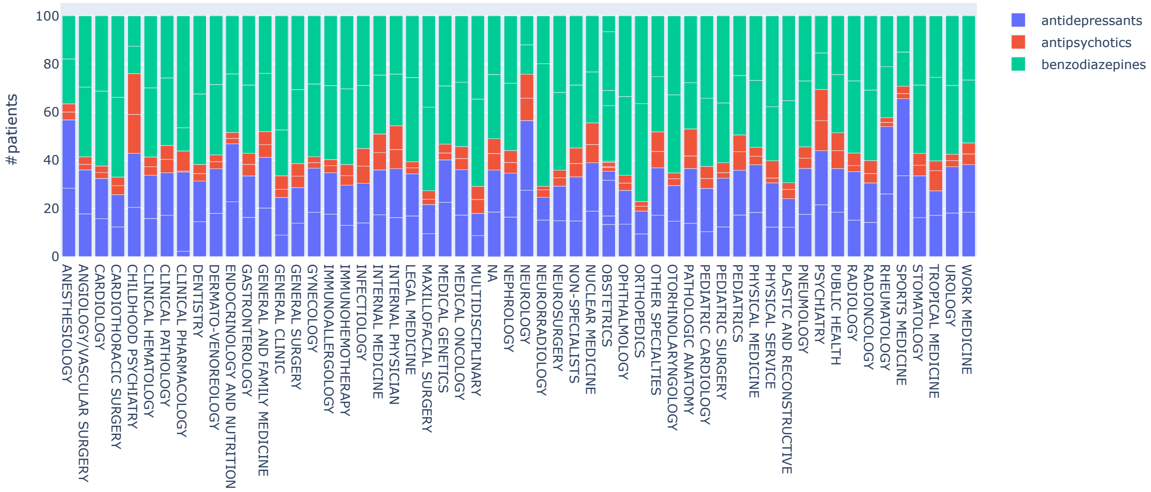

(b) number of patients

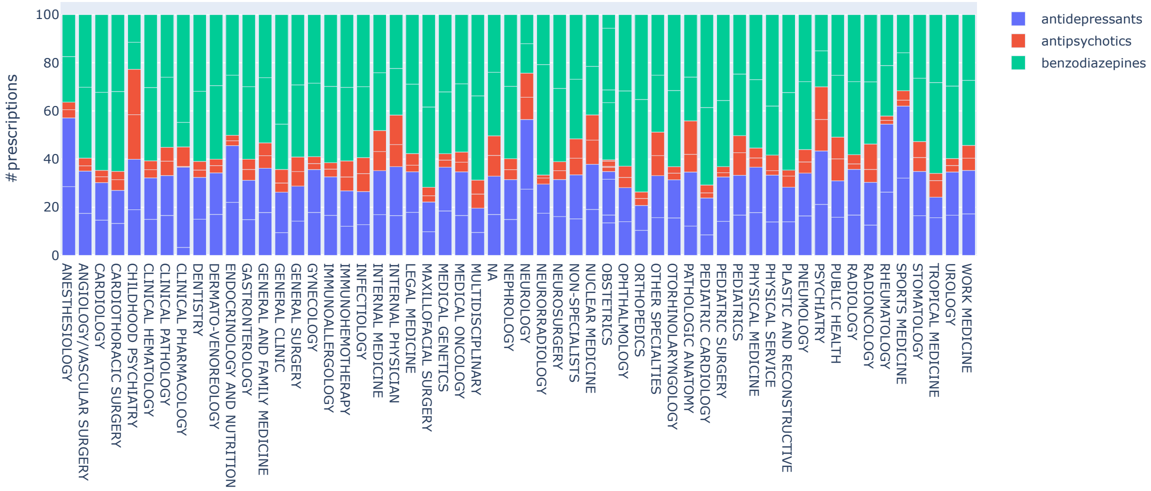

(c) prescription rate (DIDs)

Figure A13. Distribution of the prescription volume for each class of psychotropic drugs per medical specialty, 2018–2019.

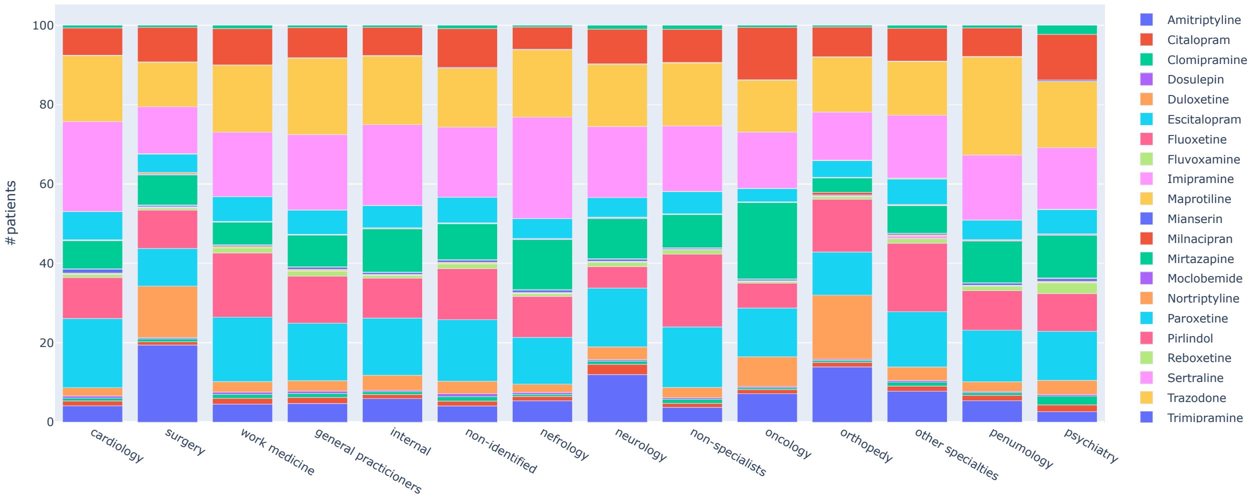

(a) antidepressants

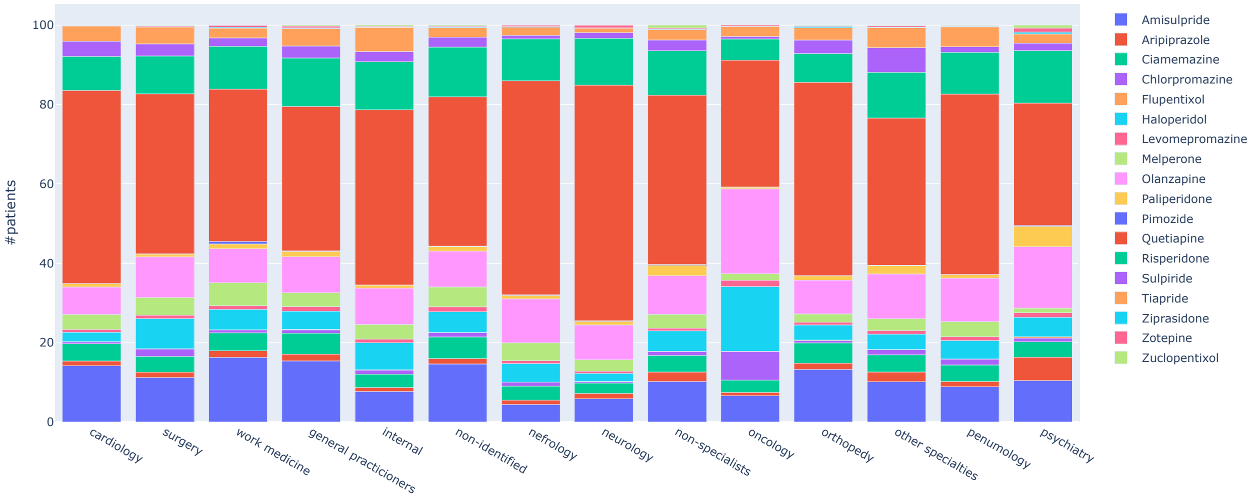

(b) antipsychotics

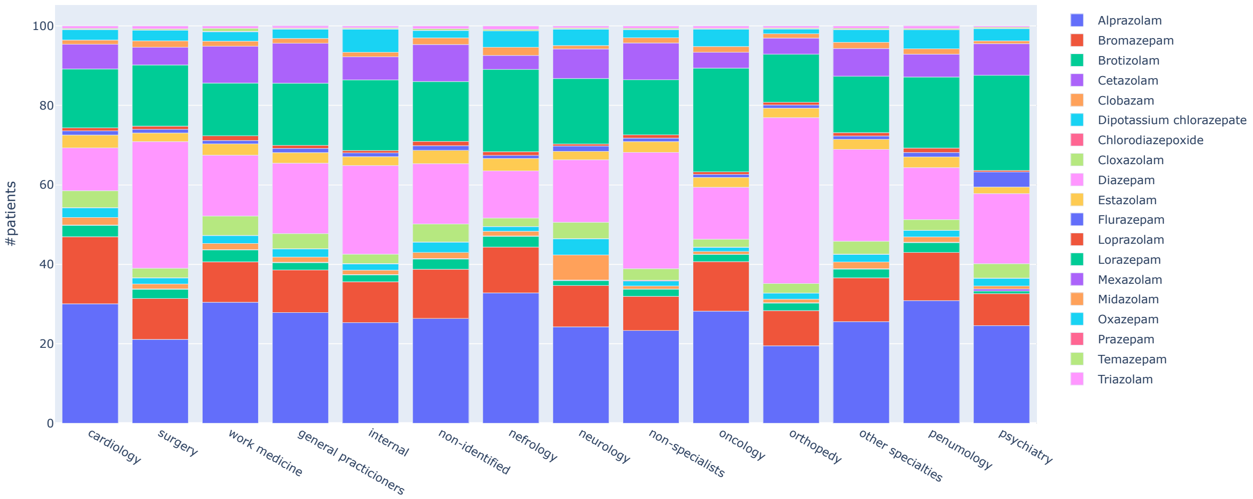

(c) benzodiazepines

Figure A14. Distribution of patients per psychotropic drug and medical specialty, 2018–2019.

B1 Psychotropic drugs

- **antidepressants**
  - selective serotonin reuptake inhibitors, including Citalopram, Escitalopram, Fluoxetine, Paroxetine, Sertraline, Fluvoxamine and Vortioxetine;
  - selective serotonin and norepinephrine reuptake inhibitors, including Milnacipran, Venlafaxine, Duloxetine;
  - tricyclic antidepressants, including Amitriptyline, Imipramine, Maprotiline, Nortriptyline, Pirlindol, Trimipramine, Clomipramine, Dosulepin;
  - alpha-2 antagonists, including Mianserin, Mirtazapine and Trazodone; and
  - others not directly falling into one of the above classes, including Moclobemide and Reboxetine.
- **antipsychotics**
  - *atypical*, including Amisulpride, Aripiprazole, Melperone, Olanzapine, Paliperidone, Quetiapine, Risperidone, Sulpiride, Tiapride, Ziprasidone, Zotepine, and Zuclopenthixol; and
  - *typical*, including Ciamemazine, Chlorpromazine, Fluphenazine, Flupentixol, Haloperidol, Levomepromazine, and Pimozide.
- **benzodiazepines**: Alprazolam, Bromazepam, Brotizolam, Cetazolam, Clobazam, Dipotassium chlorazepate, Chlorodiazepoxide (with and without clidinium bromide), Cloxazolam, Diazepam, Estazolam, Flurazepam, Loprazolam, Lorazepam, Mexazolam, Midazolam, Oxazepam, Prazepam, Temazepam and Triazolam.

B2 Dosages

Table B1. Full list of drug boxes and their corresponding ATC code, dosage, and total DDDs.

class	active ingredient	mode	box	dosage per unit	total dosage (g)	ATC code	DDD	DDDs per package
**antidepressants**	Amitriptyline	coated tablet	blister 60 unit(s)	10 mg	0.6	N06AA09	75 mg	8
	Amitriptyline	coated tablet	blister 60 unit(s)	25 mg	1.5	N06AA09	75 mg	20
	Amitriptyline	coated tablet	blister 60 unit(s)	75 mg	4.5	N06AA09	75 mg	60
	Amitriptyline	coated tablet	blister 10 unit(s)	10 mg	0.1	N06AA09	75 mg	1.33
	Amitriptyline	coated tablet	blister 20 unit(s)	10 mg	0.2	N06AA09	75 mg	2.66
	Citalopram	film coated tablet	blister 56 unit(s)	20 mg	1.12	N06AB04	20 mg	56
	Citalopram	film coated tablet	blister 60 unit(s)	20 mg	1.2	N06AB04	20 mg	60
	Citalopram	film coated tablet	blister 56 unit(s)	10 mg	0.56	N06AB04	20 mg	28
	Citalopram	film coated tablet	blister 30 unit(s)	40 mg	1.2	N06AB04	20 mg	60
	Citalopram	film coated tablet	blister 60 unit(s)	10 mg	0.6	N06AB04	20 mg	30
	Citalopram	film coated tablet	blister 28 unit(s)	40 mg	1.12	N06AB04	20 mg	56
	Citalopram	film coated tablet	blister 14 unit(s)	10 mg	0.14	N06AB04	20 mg	7
	Citalopram	film coated tablet	blister 10 unit(s)	10 mg	0.1	N06AB04	20 mg	5
	Citalopram	film coated tablet	blister 14 unit(s)	20 mg	0.28	N06AB04	20 mg	14
	Clomipramine	coated tablet	blister 60 unit(s)	25 mg	1.5	N06AA04	100 mg	15
	Clomipramine	prolonged release tablet	blister 60 unit(s)	75 mg	4.5	N06AA04	100 mg	45
	Clomipramine	coated tablet	blister 60 unit(s)	10 mg	0.6	N06AA04	100 mg	6
	Clomipramine	injectable solution	ampoule 5 unit(s) 2 ml	25 mg/2 ml	0.125	N06AA04	100 mg	1.25
	Clomipramine	coated tablet	blister 10 unit(s)	10 mg	0.1	N06AA04	100 mg	1
	Dosulepin	coated tablet	blister 60 unit(s)	75 mg	4.5	N06AA16	150 mg	30
	Dosulepin	coated tablet	blister 20 unit(s)	75 mg	1.5	N06AA16	150 mg	10
	Duloxetine	gastro-resistant capsule	blister 28 unit(s)	60 mg	1.68	N06AX21	60 mg	28
	Duloxetine	gastro-resistant capsule	blister 28 unit(s)	30 mg	0.84	N06AX21	60 mg	14
	Duloxetine	gastro-resistant capsule	blister 7 unit(s)	30 mg	0.21	N06AX21	60 mg	3.5
	Escitalopram	film coated tablet	blister 56 unit(s)	20 mg	1.12	N06AB10	10 mg	112
	Escitalopram	film coated tablet	blister 56 unit(s)	10 mg	0.56	N06AB10	10 mg	56
	Escitalopram	film coated tablet	blister 60 unit(s)	10 mg	0.6	N06AB10	10 mg	60
	Escitalopram	orodispersible tablet	blister 56 unit(s)	10 mg	0.56	N06AB10	10 mg	56
	Escitalopram	orodispersible tablet	blister 56 unit(s)	20 mg	1.12	N06AB10	10 mg	112
	Escitalopram	film coated tablet	blister 14 unit(s)	10 mg	0.14	N06AB10	10 mg	14
	Escitalopram	oral drops - solution	dropper bottle 1x 15 ml	20 mg/ml	0.3	N06AB10	10 mg	30
	Fluoxetine	capsule	blister 60 unit(s)	20 mg	1.2	N06AB03	20 mg	60
	Fluoxetine	capsule	blister 56 unit(s)	20 mg	1.12	N06AB03	20 mg	56
	Fluoxetine	oral solution	bottle 1x 140 ml	4 mg/ml	0.56	N06AB03	20 mg	28
	Fluoxetine	capsule	blister 10 unit(s)	20 mg	0.2	N06AB03	20 mg	10
	Fluoxetine	capsule	blister 20 unit(s)	20 mg	0.4	N06AB03	20 mg	20
	Fluoxetine	capsule	blister 14 unit(s)	20 mg	0.28	N06AB03	20 mg	14
	Fluoxetine	capsule	blister 50 unit(s)	20 mg	1	N06AB03	20 mg	50
	Fluoxetine	capsule	blister 28 unit(s)	20 mg	0.56	N06AB03	20 mg	28
	Fluvoxamine	film coated tablet	blister 60 unit(s)	50 mg	3	N06AB08	100 mg	30
	Fluvoxamine	film coated tablet	blister 60 unit(s)	100 mg	6	N06AB08	100 mg	60
	Fluvoxamine	film coated tablet	blister 10 unit(s)	50 mg	0.5	N06AB08	100 mg	5
	Fluvoxamine	film coated tablet	blister 20 unit(s)	50 mg	1	N06AB08	100 mg	10
	Imipramine	coated tablet	blister 60 unit(s)	25 mg	1.5	N06AA02	100 mg	15
	Imipramine	coated tablet	blister 60 unit(s)	10 mg	0.6	N06AA02	100 mg	6
	Maprotiline	film coated tablet	blister 60 unit(s)	75 mg	4.5	N06AA21	100 mg	45
	Maprotiline	coated tablet	blister 60 unit(s)	50 mg	3	N06AA21	100 mg	30
	Maprotiline	coated tablet	blister 60 unit(s)	25 mg	1.5	N06AA21	100 mg	15
	Maprotiline	film coated tablet	blister 60 unit(s)	50 mg	3	N06AA21	100 mg	30
	Maprotiline	coated tablet	blister 60 unit(s)	75 mg	4.5	N06AA21	100 mg	45
	Maprotiline	coated tablet	blister 10 unit(s)	25 mg	0.25	N06AA21	100 mg	2.5
	Maprotiline	film coated tablet	blister 60 unit(s)	25 mg	1.5	N06AA21	100 mg	15
	Mianserin	coated tablet	blister 10 unit(s)	30 mg	0.3	N06AX03	60 mg	5
	Mianserin	coated tablet	blister 60 unit(s)	30 mg	1.8	N06AX03	60 mg	30
	Milnacipran	capsule	blister 56 unit(s)	25 mg	1.4	N06AX17	100 mg	14
	Milnacipran	capsule	blister 56 unit(s)	50 mg	2.8	N06AX17	100 mg	28
	Mirtazapine	orodispersible tablet	blister 30 unit(s)	15 mg	0.45	N06AX11	30 mg	15
	Mirtazapine	film coated tablet	blister 30 unit(s)	15 mg	0.45	N06AX11	30 mg	15
	Mirtazapine	orodispersible tablet	blister 60 unit(s)	15 mg	0.9	N06AX11	30 mg	30
	Mirtazapine	film coated tablet	blister 60 unit(s)	30 mg	1.8	N06AX11	30 mg	60
	Mirtazapine	orodispersible tablet	blister 60 unit(s)	30 mg	1.8	N06AX11	30 mg	60
	Mirtazapine	coated tablet	blister 30 unit(s)	30 mg	0.9	N06AX11	30 mg	30
	Mirtazapine	orodispersible tablet	blister 30 unit(s)	30 mg	0.9	N06AX11	30 mg	30
	Mirtazapine	film coated tablet	blister 30 unit(s)	30 mg	0.9	N06AX11	30 mg	30
	Mirtazapine	coated tablet	blister 56 unit(s)	15 mg	0.84	N06AX11	30 mg	28
	Mirtazapine	coated tablet	blister 56 unit(s)	30 mg	1.68	N06AX11	30 mg	56
	Mirtazapine	film coated tablet	blister 56 unit(s)	15 mg	0.84	N06AX11	30 mg	28
	Mirtazapine	film coated tablet	blister 60 unit(s)	15 mg	0.9	N06AX11	30 mg	30
	Mirtazapine	orodispersible tablet	blister 30 unit(s)	45 mg	1.35	N06AX11	30 mg	45
	Mirtazapine	film coated tablet	blister 56 unit(s)	30 mg	1.68	N06AX11	30 mg	56
	Mirtazapine	orodispersible tablet	blister 18 unit(s)	15 mg	0.27	N06AX11	30 mg	9
	Mirtazapine	coated tablet	blister 14 unit(s)	15 mg	0.21	N06AX11	30 mg	7
	Mirtazapine	orodispersible tablet	blister 20 unit(s)	15 mg	0.3	N06AX11	30 mg	10
	Mirtazapine	orodispersible tablet	blister 20 unit(s)	30 mg	0.6	N06AX11	30 mg	20
	Mirtazapine	film coated tablet	blister 10 unit(s)	15 mg	0.15	N06AX11	30 mg	5
	Mirtazapine	film coated tablet	blister 20 unit(s)	15 mg	0.3	N06AX11	30 mg	10
	Mirtazapine	orodispersible tablet	blister 6 unit(s)	15 mg	0.09	N06AX11	30 mg	3
	Mirtazapine	film coated tablet	blister 20 unit(s)	30 mg	0.6	N06AX11	30 mg	20
	Mirtazapine	film coated tablet	blister 14 unit(s)	15 mg	0.21	N06AX11	30 mg	7
	Mirtazapine	film coated tablet	blister 10 unit(s)	30 mg	0.3	N06AX11	30 mg	10
	Mirtazapine	film coated tablet	blister 15 unit(s)	30 mg	0.45	N06AX11	30 mg	15

class	active drug	mode	box	dosage per unit	total dosage (g)	ATC code	DDD	DDDs per package
**antidepressants**	Mirtazapine	film coated tablet	blister 15 unit(s)	15 mg	0.225	N06AX11	30 mg	7.5
	Mirtazapine	coated tablet	blister 10 unit(s)	30 mg	0.3	N06AX11	30 mg	10
	Mirtazapine	film coated tablet	blister 14 unit(s)	30 mg	0.42	N06AX11	30 mg	14
	Mirtazapine	orodispersible tablet	thermowelded tape 30 units	30 mg	0.9	N06AX11	30 mg	30
	Mirtazapine	orodispersible tablet	thermowelded tape 30 units	15 mg	0.45	N06AX11	30 mg	15
	Mirtazapine	orodispersible tablet	thermowelded tape 6 unit(s)	15 mg	0.09	N06AX11	30 mg	3
	Mirtazapine	film coated tablet	blister 28 unit(s)	30 mg	0.84	N06AX11	30 mg	28
	Mirtazapine	film coated tablet	blister 28 unit(s)	15 mg	0.42	N06AX11	30 mg	14
	Moclobemide	film coated tablet	blister 60 unit(s)	150 mg	9	N06AG02	300 mg	30
	Moclobemide	film coated tablet	blister 60 unit(s)	300 mg	18	N06AG02	300 mg	60
	Moclobemide	film coated tablet	blister 20 unit(s)	150 mg	3	N06AG02	300 mg	10
	Moclobemide	film coated tablet	blister 10 unit(s)	150 mg	1.5	N06AG02	300 mg	5
	Nortriptyline	coated tablet	blister 60 unit(s)	25 mg	1.5	N06AA10	75 mg	20
	Nortriptyline	coated tablet	blister 20 unit(s)	25 mg	0.5	N06AA10	75 mg	6.67
	Paroxetine	pill	blister 60 unit(s)	20 mg	1.2	N06AB05	20 mg	60
	Paroxetine	film coated tablet	blister 60 unit(s)	20 mg	1.2	N06AB05	20 mg	60
	Paroxetine	film coated tablet	bottle 60 unit(s)	20 mg	1.2	N06AB05	20 mg	60
	Paroxetine	oral drops - solution	bottle 1x 30 ml	10 mg/ml	0.3	N06AB05	20 mg	15
	Paroxetine	film coated tablet	blister 20 unit(s)	20 mg	0.4	N06AB05	20 mg	20
	Paroxetine	film coated tablet	blister 10 unit(s)	20 mg	0.2	N06AB05	20 mg	10
	Paroxetine	film coated tablet	bottle 10 unit(s)	20 mg	0.2	N06AB05	20 mg	10
	Paroxetine	pill	blister 10 unit(s)	20 mg	0.2	N06AB05	20 mg	10
	Paroxetine	film coated tablet	bottle 30 unit(s)	20 mg	0.6	N06AB05	20 mg	30
	Paroxetine	film coated tablet	blister 30 unit(s)	20 mg	0.6	N06AB05	20 mg	30
	Paroxetine	pill	blister 20 unit(s)	20 mg	0.4	N06AB05	20 mg	20
	Pirlindol	pill	bottle 60 unit(s)	50 mg	3	-	200 mg	15
	Pirlindol	pill	blister 60 unit(s)	100 mg	6	-	200 mg	30
	Pirlindol	pill	blister 60 unit(s)	75 mg	4.5	-	200 mg	22.5
	Pirlindol	pill	bottle 20 unit(s)	50 mg	1	-	200 mg	5
	Reboxetine	pill	blister 60 unit(s)	4 mg	0.24	N06AX18	8 mg	30
	Reboxetine	pill	blister 20 unit(s)	4 mg	0.08	N06AX18	8 mg	10
	Sertraline	film coated tablet	blister 60 unit(s)	50 mg	3	N06AB06	50 mg	60
	Sertraline	film coated tablet	blister 56 unit(s)	50 mg	2.8	N06AB06	50 mg	56
	Sertraline	film coated tablet	blister 60 unit(s)	100 mg	6	N06AB06	50 mg	120
	Sertraline	film coated tablet	blister 56 unit(s)	100 mg	5.6	N06AB06	50 mg	112
	Sertraline	film coated tablet	blister 20 unit(s)	50 mg	1	N06AB06	50 mg	20
	Sertraline	film coated tablet	blister 10 unit(s)	50 mg	0.5	N06AB06	50 mg	10
	Sertraline	film coated tablet	blister 14 unit(s)	50 mg	0.7	N06AB06	50 mg	14
	Sertraline	concentrate for oral solution	bottle 1x 60 ml	20 mg/ml	1.2	N06AB06	50 mg	24
	Sertraline	film coated tablet	blister 30 unit(s)	50 mg	1.5	N06AB06	50 mg	30
	Sertraline	film coated tablet	blister 30 unit(s)	100 mg	3	N06AB06	50 mg	60
	Trazodone	modified release tablet	blister 60 unit(s)	150 mg	9	N06AX05	300 mg	30
	Trazodone	modified release tablet	blister 20 unit(s)	150 mg	3	N06AX05	300 mg	10
	Trazodone	film coated tablet	blister 60 unit(s)	100 mg	6	N06AX05	300 mg	20
	Trazodone	prolonged release tablet	blister 30 unit(s)	300 mg	9	N06AX05	300 mg	30
	Trazodone	pill	blister 60 unit(s)	150 mg	9	N06AX05	300 mg	30
	Trazodone	pill	blister 60 unit(s)	100 mg	6	N06AX05	300 mg	20
	Trazodone	prolonged release tablet	blister 10 unit(s)	150 mg	1.5	N06AX05	300 mg	5
	Trazodone	film coated tablet	blister 20 unit(s)	100 mg	2	N06AX05	300 mg	6.67
	Trazodone	pill	blister 20 unit(s)	100 mg	2	N06AX05	300 mg	6.67
	Trazodone	pill	blister 20 unit(s)	150 mg	3	N06AX05	300 mg	10
	Trimipramine	pill	blister 60 unit(s)	25 mg	1.5	N06AA06	150 mg	10
	Trimipramine	coated tablet	blister 60 unit(s)	100 mg	6	N06AA06	150 mg	40
	Trimipramine	pill	blister 10 unit(s)	25 mg	0.25	N06AA06	150 mg	1.67
	Venlafaxine	prolonged release tablet	blister 30 unit(s)	150 mg	4.5	N06AX16	100 mg	45
	Venlafaxine	prolonged release tablet	blister 30 unit(s)	37.5 mg	1.125	N06AX16	100 mg	11.25
	Venlafaxine	extended release capsule	blister 30 unit(s)	75 mg	2.25	N06AX16	100 mg	22.5
	Venlafaxine	extended release capsule	blister 30 unit(s)	150 mg	4.5	N06AX16	100 mg	45
	Venlafaxine	prolonged release tablet	blister 30 unit(s)	225 mg	6.75	N06AX16	100 mg	67.5
	Venlafaxine	film coated tablet	blister 60 unit(s)	75 mg	4.5	N06AX16	100 mg	45
	Venlafaxine	prolonged release tablet	blister 30 unit(s)	75 mg	2.25	N06AX16	100 mg	22.5
	Venlafaxine	extended release capsule	blister 30 unit(s)	37.5 mg	1.125	N06AX16	100 mg	11.25
	Venlafaxine	extended release capsule	blister 30 unit(s)	225 mg	6.75	N06AX16	100 mg	67.5
	Venlafaxine	film coated tablet	blister 60 unit(s)	37.5 mg	2.25	N06AX16	100 mg	22.5
	Venlafaxine	extended release capsule	blister 10 unit(s)	75 mg	0.75	N06AX16	100 mg	7.5
	Venlafaxine	prolonged release tablet	blister 10 unit(s)	37.5 mg	0.375	N06AX16	100 mg	3.75
	Venlafaxine	extended release capsule	blister 10 unit(s)	37.5 mg	0.375	N06AX16	100 mg	3.75
	Venlafaxine	prolonged release tablet	blister 10 unit(s)	75 mg	0.75	N06AX16	100 mg	7.5
	Venlafaxine	extended release capsule	blister 20 unit(s)	37.5 mg	0.75	N06AX16	100 mg	7.5
	Venlafaxine	film coated tablet	blister 10 unit(s)	37.5 mg	0.375	N06AX16	100 mg	3.75
	Venlafaxine	oral solution	bottle 1x 60 ml	75 mg/ml	4.5	N06AX16	100 mg	45
	Vortioxetine	film coated tablet	blister 28 unit(s)	10 mg	0.28	N06AX26	10 mg	28
	Vortioxetine	film coated tablet	blister 28 unit(s)	20 mg	0.56	N06AX26	10 mg	56
	Vortioxetine	film coated tablet	blister 28 unit(s)	5 mg	0.14	N06AX26	10 mg	14
	Vortioxetine	film coated tablet	blister 14 unit(s)	5 mg	0.07	N06AX26	10 mg	7
	Vortioxetine	oral drops - solution	bottle 1x 15 ml	20 mg/ml	0.3	N06AX26	10 mg	30
	Vortioxetine	film coated tablet	blister 28 unit(s)	15 mg	0.42	N06AX26	10 mg	42
**antipsychotics**	Amissulpride	oral solution	ampoule 20 unit(s) 10 ml	50 mg/10 ml	1	N05AL05	0.4 g	2.5
	Amissulpride	pill	blister 60 unit(s)	50 mg	3	N05AL05	0.4 g	7.5
	Amissulpride	pill	blister 20 unit(s)	50 mg	1	N05AL05	0.4 g	2.5
	Amissulpride	pill	blister 60 unit(s)	200 mg	12	N05AL05	0.4 g	30
	Aripiprazole	pill	blister 28 unit(s)	10 mg	0.28	N05AX12	15 mg	18.67
	Aripiprazole	prolonged-release injection	vial 1x 1.9 ml	400 mg	0	N05AX12	13,3 mg	30.08
	Aripiprazole	pill	blister 28 unit(s)	15 mg	0.42	N05AX12	15 mg	28
	Aripiprazole	pill	blister 28 unit(s)	5 mg	0.14	N05AX12	15 mg	9.33
	Aripiprazole	oral solution	bottle 1x 150 ml	1 mg/ml	0.15	N05AX12	15 mg	10
	Aripiprazole	pill	blister 14 unit(s)	10 mg	0.14	N05AX12	15 mg	9.33
	Aripiprazole	pill	blister 14 unit(s)	5 mg	0.07	N05AX12	15 mg	4.67
	Aripiprazole	pill	blister 14 unit(s)	15 mg	0.21	N05AX12	15 mg	14
	Aripiprazole	injectable solution	vial 1x 1.3 ml	7.5 mg/ml	0	N05AX12	15 mg	0.65
	Ciamemazine	oral drops - solution	bottle 1x 30 ml	40 mg/ml	1.2	N05AA06	100 mg	12
	Ciamemazine	coated tablet	blister 60 unit(s)	100 mg	6	N05AA06	100 mg	60
	Ciamemazine	coated tablet	blister 20 unit(s)	100 mg	2	N05AA06	100 mg	20
	Cloropromazine	coated tablet	blister 20 unit(s)	25 mg	0.5	N05AA01	300 mg	1.67
	Cloropromazine	coated tablet	blister 60 unit(s)	25 mg	1.5	N05AA01	300 mg	5
	Cloropromazine	coated tablet	blister 60 unit(s)	100 mg	6	N05AA01	300 mg	20
	Cloropromazine	oral drops - solution	dropper bottle 1x 30 ml	40 mg/ml	1.2	N05AA01	300 mg	4
	Cloropromazine	injectable solution	ampoule 5 unit(s) 2 ml	50 mg/2 ml	0.25	N05AA01	100 mg	2.5
	Cloropromazine	injectable solution	ampoule 6 unit(s) 5 ml	25 mg/5 ml	0.15	N05AA01	100 mg	1.5
	Clorpromazina	coated tablet	blister 60 unit(s)	100 mg	6	N05AA01	300 mg	20
	Clorpromazina	coated tablet	blister 60 unit(s)	25 mg	1.5	N05AA01	300 mg	5
	Clorpromazina	coated tablet	blister 20 unit(s)	25 mg	0.5	N05AA01	300 mg	1.67
	Clorpromazina	oral drops - solution	dropper bottle 1x 30 ml	40 mg/ml	1.2	N05AA01	300 mg	4
	Clorpromazina	injectable solution	ampoule 6 unit(s) 5 ml	25 mg/5 ml	0.15	N05AA01	100 mg	1.5
	Clorpromazina	injectable solution	ampoule 5 unit(s) 2 ml	50 mg/2 ml	0.25	N05AA01	100 mg	2.5
	Flufenazine	injectable solution	ampoule 1x 1 ml	25 mg/ml	0.025	N05AB02	1 mg	25
	Flupentixol	injectable solution	ampoule 1x 1 ml	100 mg/1 ml	0.1	N05AF01	4 mg	25
	Flupentixol	injectable solution	ampoule 5 unit(s) 1 ml	20 mg/1 ml	0.1	N05AF01	4 mg	25
	Haloperidol	oral solution	bottle 1x 30 ml	2 mg/ml	0.06	N05AD01	8 mg	7.5
	Haloperidol	pill	blister 60 unit(s)	1 mg	0.06	N05AD01	8 mg	7.5
	Haloperidol	pill	blister 60 unit(s)	5 mg	0.3	N05AD01	8 mg	37.5
	Haloperidol	injectable solution	ampoule 1x 1 ml	100 mg/1 ml	0.1	N05AD01	3.3 mg	30.3
	Haloperidol	injectable solution	ampoule 1x 1 ml	50 mg/1 ml	0.05	N05AD01	3.3 mg	15.15
	Haloperidol	pill	blister 60 unit(s)	10 mg	0.6	N05AD01	8 mg	75
	Haloperidol	pill	blister 20 unit(s)	1 mg	0.02	N05AD01	8 mg	2.5
	Haloperidol	injectable solution	ampoule 5 unit(s) 1 ml	5 mg/1 ml	0.025	N05AD01	8 mg	3.125
	Haloperidol	pill	blister 60 unit(s)	2 mg	0.12	N05AD01	8 mg	15
	Levomepromazine	oral drops - solution	bottle 1x 30 ml	40 mg/ml	1.2	N05AA02	300 mg	4
	Levomepromazine	coated tablet	blister 60 unit(s)	100 mg	6	N05AA02	300 mg	20
	Levomepromazine	coated tablet	blister 60 unit(s)	25 mg	1.5	N05AA02	300 mg	5
	Levomepromazine	coated tablet	blister 20 unit(s)	25 mg	0.5	N05AA02	300 mg	1.67
	Levomepromazine	injectable solution	ampoule 6 unit(s) 1 ml	25 mg/1 ml	0.15	N05AA02	100 mg	1.5
	Melperone	film coated tablet	bottle 60 unit(s)	25 mg	1.5	N05AD03	300 mg	5
	Melperone	film coated tablet	bottle 60 unit(s)	50 mg	3	N05AD03	300 mg	10
	Melperone	film coated tablet	bottle 20 unit(s)	25 mg	0.5	N05AD03	300 mg	1.67
	Olanzapine	film coated tablet	blister 28 unit(s)	2.5 mg	0.07	N05AH03	10 mg	7
	Olanzapine	orodispersible tablet	blister 28 unit(s)	10 mg	0.28	N05AH03	10 mg	28
	Olanzapine	pill	blister 28 unit(s)	5 mg	0.14	N05AH03	10 mg	14
	Olanzapine	pill	blister 28 unit(s)	10 mg	0.28	N05AH03	10 mg	28
	Olanzapine	orodispersible tablet	blister 28 unit(s)	5 mg	0.14	N05AH03	10 mg	14
	Olanzapine	film coated tablet	blister 28 unit(s)	10 mg	0.28	N05AH03	10 mg	28
	Olanzapine	pill	blister 28 unit(s)	2.5 mg	0.07	N05AH03	10 mg	7
	Olanzapine	coated tablet	blister 28 unit(s)	2.5 mg	0.07	N05AH03	10 mg	7
	Olanzapine	coated tablet	blister 28 unit(s)	5 mg	0.14	N05AH03	10 mg	14
	Olanzapine	film coated tablet	blister 28 unit(s)	5 mg	0.14	N05AH03	10 mg	14
	Olanzapine	coated tablet	blister 28 unit(s)	10 mg	0.28	N05AH03	10 mg	28
	Olanzapine	orodispersible tablet	blister 28 unit(s)	20 mg	0.56	N05AH03	10 mg	56
	Olanzapine	pill	blister 28 unit(s)	15 mg	0.42	N05AH03	10 mg	42
	Olanzapine	orodispersible tablet	blister 28 unit(s)	15 mg	0.42	N05AH03	10 mg	42
	Olanzapine	film coated tablet	blister 28 unit(s)	7.5 mg	0.21	N05AH03	10 mg	21
	Olanzapine	pill	blister 28 unit(s)	7.5 mg	0.21	N05AH03	10 mg	21
	Olanzapine	coated tablet	blister 28 unit(s)	15 mg	0.42	N05AH03	10 mg	42
	Olanzapine	coated tablet	blister 28 unit(s)	7.5 mg	0.21	N05AH03	10 mg	21
	Olanzapine	film coated tablet	blister 28 unit(s)	15 mg	0.42	N05AH03	10 mg	42
	Olanzapine	film coated tablet	blister 14 unit(s)	5 mg	0.07	N05AH03	10 mg	7
	Olanzapine	pill	blister 14 unit(s)	2.5 mg	0.035	N05AH03	10 mg	3.5
	Paliperidone	prolonged release injection	pre-filled syringe 1x	150 mg	0.15	N05AX13	2.5 mg	60
	Paliperidone	prolonged release tablet	blister 28 unit(s)	3 mg	0.084	N05AX13	6 mg	14
	Paliperidone	prolonged release injection	pre-filled syringe 1x	100 mg	0.1	N05AX13	2.5 mg	40
	Paliperidone	prolonged release tablet	blister 28 unit(s)	6 mg	0.168	N05AX13	6 mg	28
	Paliperidone	prolonged release tablet	blister 28 unit(s)	9 mg	0.252	N05AX13	6 mg	42
	Paliperidone	prolonged release injection	pre-filled syringe 1x 0.875 ml	175 mg	0	N05AX13	2.5 mg	70
	Paliperidone	prolonged release injection	pre-filled syringe 1x 2.625 ml	525 mg	0	N05AX13	2.5 mg	210
	Paliperidone	prolonged release injection	pre-filled syringe 1x 1.75 ml	350 mg	0	N05AX13	2.5 mg	140
	Paliperidone	prolonged release injection	pre-filled syringe 1x	50 mg	0.05	N05AX13	2.5 mg	20
	Paliperidone	prolonged release injection	pre-filled syringe 1x	75 mg	0.075	N05AX13	2.5 mg	30
	Paliperidone	prolonged release injection	pre-filled syringe 1x 1.315 ml	263 mg	0	N05AX13	2.5 mg	105.2
	Paliperidone	prolonged release tablet	blister 14 unit(s)	3 mg	0.042	N05AX13	6 mg	7
	Pimozide	pill	blister 60 unit(s)	4 mg	0.24	N05AG02	4 mg	60
	Pimozide	pill	blister 20 unit(s)	4 mg	0.08	N05AG02	4 mg	20
	Quetiapine	prolonged release tablet	blister 60 unit(s)	50 mg	3	N05AH04	400 mg	7.5
	Quetiapine	film coated tablet	blister 20 unit(s)	25 mg	0.5	N05AH04	400 mg	1.25
	Quetiapine	film coated tablet	blister 60 unit(s)	100 mg	6	N05AH04	400 mg	15
	Quetiapine	prolonged release tablet	blister 60 unit(s)	300 mg	18	N05AH04	400 mg	45
	Quetiapine	prolonged release tablet	blister 30 unit(s)	150 mg	4.5	N05AH04	400 mg	11.25

class	active drug	mode	box	dosage per unit	total dosage (g)	ATC code	DDD	DDDs per package
**antipsychotics**	Quetiapine	film coated tablet	blister 60 unit(s)	200 mg	12	N05AH04	400 mg	30
	Quetiapine	prolonged release tablet	blister 60 unit(s)	200 mg	12	N05AH04	400 mg	30
	Quetiapine	film coated tablet	blister 60 unit(s)	300 mg	18	N05AH04	400 mg	45
	Quetiapine	prolonged release tablet	blister 60 unit(s)	400 mg	24	N05AH04	400 mg	60
	Quetiapine	film coated tablet	blister 10 unit(s)	25 mg	0.25	N05AH04	400 mg	0.625
	Quetiapine	prolonged release tablet	blister 10 unit(s)	50 mg	0.5	N05AH04	400 mg	1.25
	Quetiapine	prolonged release tablet	blister 10 unit(s)	200 mg	2	N05AH04	400 mg	5
	Quetiapine	film coated tablet	blister 6 unit(s)	25 mg	0.15	N05AH04	400 mg	0.375
	Quetiapine	prolonged release tablet	blister 10 unit(s)	300 mg	3	N05AH04	400 mg	7.5
	Quetiapine	film coated tablet	blister 10 unit(s)	25+100+200mg	0.65	N05AH04	400 mg	1.625
	Quetiapine	prolonged release tablet	bottle 10 unit(s)	200 mg	2	N05AH04	400 mg	5
	Risperidone	film coated tablet	blister 60 unit(s)	0.5 mg	0.03	N05AX08	5 mg	6
	Risperidone	oral solution	bottle 1x 30 ml	1 mg/ml	0.03	N05AX08	5 mg	6
	Risperidone	film coated tablet	blister 60 unit(s)	2 mg	0.12	N05AX08	5 mg	24
	Risperidone	film coated tablet	blister 60 unit(s)	1 mg	0.06	N05AX08	5 mg	12
	Risperidone	prolonged release injection	vial 1x 2 ml	37.5mg/2ml	0.0375	N05AX08	2.7 mg	13.89
	Risperidone	film coated tablet	blister 60 unit(s)	3 mg	0.18	N05AX08	5 mg	36
	Risperidone	prolonged release injection	vial 1x 2 ml	25 mg/2 ml	0.025	N05AX08	2.7 mg	9.26
	Risperidone	orodispersible tablet	blister 60 unit(s)	0.5 mg	0.03	N05AX08	5 mg	6
	Risperidone	orodispersible tablet	blister 60 unit(s)	1 mg	0.06	N05AX08	5 mg	12
	Risperidone	prolonged release injection	vial 1x 2 ml	50 mg/2 ml	0.05	N05AX08	2.7 mg	18.52
	Risperidone	orodispersible tablet	blister 60 unit(s)	2 mg	0.12	N05AX08	5 mg	24
	Risperidone	orodispersible tablet	blister 28 unit(s)	2 mg	0.056	N05AX08	5 mg	11.2
	Risperidone	film coated tablet	blister 60 unit(s)	4 mg	0.24	N05AX08	5 mg	48
	Risperidone	film coated tablet	blister 20 unit(s)	0.5 mg	0.01	N05AX08	5 mg	2
	Risperidone	orodispersible tablet	blister 56 unit(s)	3 mg	0.168	N05AX08	5 mg	33.6
	Risperidone	orodispersible tablet	blister 56 unit(s)	2 mg	0.112	N05AX08	5 mg	22.4
	Risperidone	orodispersible tablet	blister 20 unit(s)	0.5 mg	0.01	N05AX08	5 mg	2
	Risperidone	film coated tablet	blister 20 unit(s)	1 mg	0.02	N05AX08	5 mg	4
	Risperidone	orodispersible tablet	blister 56 unit(s)	4 mg	0.224	N05AX08	5 mg	44.8
	Risperidone	orodispersible tablet	blister 20 unit(s)	1 mg	0.02	N05AX08	5 mg	4
	Risperidone	orodispersible tablet	blister 14 unit(s)	2 mg	0.028	N05AX08	5 mg	5.6
	Risperidone	orodispersible tablet	blister 14 unit(s)	0.5 mg	0.007	N05AX08	5 mg	1.4
	Risperidone	orodispersible tablet	blister 56 unit(s)	0.5 mg	0.028	N05AX08	5 mg	5.6
	Risperidone	orodispersible tablet	blister 56 unit(s)	1 mg	0.056	N05AX08	5 mg	11.2
	Risperidone	powder injectable suspension	vial 1x 2 ml	25 mg/2 ml	0.025	N05AX08	2.7 mg	9.26
	Risperidone	powder injectable suspension	vial 1x 2 ml	50 mg/2 ml	0.05	N05AX08	2.7 mg	18.52
	Risperidone	powder injectable suspension	vial 1x 2 ml	37.5mg/2ml	0.0375	N05AX08	2.7 mg	13.89
	Sulpiride	pill	blister 60 unit(s)	200 mg	12	N05AL01	800 mg	15
	Sulpiride	capsule	blister 60 unit(s)	50 mg	3	N05AL01	800 mg	3.75
	Sulpiride	capsule	blister 20 unit(s)	50 mg	1	N05AL01	800 mg	1.25
	Tiapride	pill	blister 60 unit(s)	100 mg	6	N05AL03	400 mg	15
	Tiapride	pill	blister 20 unit(s)	100 mg	2	N05AL03	400 mg	5
	Tiapride	injectable solution	ampoule 6 unit(s) 2 ml	100 mg/2 ml	0.6	N05AL03	400 mg	1.5
	Ziprasidone	capsule	blister 56 unit(s)	60 mg	3.36	N05AE04	80 mg	42
	Ziprasidone	capsule	blister 56 unit(s)	40 mg	2.24	N05AE04	80 mg	28
	Ziprasidone	capsule	blister 56 unit(s)	80 mg	4.48	N05AE04	80 mg	56
	Ziprasidone	capsule	blister 56 unit(s)	20 mg	1.12	N05AE04	80 mg	14
	Ziprasidone	capsule	blister 14 unit(s)	40 mg	0.56	N05AE04	80 mg	7
	Ziprasidone	capsule	blister 14 unit(s)	20 mg	0.28	N05AE04	80 mg	3.5
	Zotepine	coated tablet	blister 60 unit(s)	100 mg	6	N05AX11	200 mg	30
	Zotepine	coated tablet	blister 60 unit(s)	25 mg	1.5	N05AX11	200 mg	7.5
	Zotepine	coated tablet	blister 60 unit(s)	50 mg	3	N05AX11	200 mg	15
	Zotepine	coated tablet	blister 20 unit(s)	25 mg	0.5	N05AX11	200 mg	2.5
	Zuclopentixol	injectable solution	ampoule 1x 1 ml	200 mg/1 ml	0.2	N05AF05	30 mg	6.67
	Zuclopentixol	injectable solution	ampoule 1x 1 ml	50 mg/1 ml	0.05	N05AF05	30 mg	1.67
**benzodiazepines**	Alprazolam	pill	blister 60 unit(s)	0.5 mg	0.03	N05BA12	1 mg	30
	Alprazolam	modified release tablet	blister 60 unit(s)	1 mg	0.06	N05BA12	1 mg	60
	Alprazolam	pill	blister 60 unit(s)	0.25 mg	0.015	N05BA12	1 mg	15
	Alprazolam	pill	blister 60 unit(s)	1 mg	0.06	N05BA12	1 mg	60
	Alprazolam	modified release tablet	blister 60 unit(s)	2 mg	0.12	N05BA12	1 mg	120
	Alprazolam	modified release tablet	blister 60 unit(s)	0.5 mg	0.03	N05BA12	1 mg	30
	Alprazolam	pill	blister 20 unit(s)	0.25 mg	0.005	N05BA12	1 mg	5
	Alprazolam	pill	blister 20 unit(s)	1 mg	0.02	N05BA12	1 mg	20
	Alprazolam	pill	blister 20 unit(s)	0.5 mg	0.01	N05BA12	1 mg	10
	Alprazolam	modified release tablet	blister 20 unit(s)	1 mg	0.02	N05BA12	1 mg	20
	Alprazolam	modified release tablet	blister 20 unit(s)	0.5 mg	0.01	N05BA12	1 mg	10
	Alprazolam	modified release tablet	blister 60 unit(s)	3 mg	0.18	N05BA12	1 mg	180
	Alprazolam	modified release tablet	blister 40 unit(s)	0.5 mg	0.02	N05BA12	1 mg	20
	Alprazolam	modified release tablet	blister 40 unit(s)	1 mg	0.04	N05BA12	1 mg	40
	Alprazolam	modified release tablet	blister 20 unit(s)	2 mg	0.04	N05BA12	1 mg	40
	Alprazolam	modified release tablet	blister 20 unit(s)	3 mg	0.06	N05BA12	1 mg	60
	Alprazolam	sublingual tablet	blister 10 unit(s)	0.5 mg	0.005	N05BA12	1 mg	5
	Alprazolam	sublingual tablet	blister 20 unit(s)	0.5 mg	0.01	N05BA12	1 mg	10
	Alprazolam	sublingual tablet	blister 60 unit(s)	0.5 mg	0.03	N05BA12	1 mg	30
	Alprazolam	sublingual tablet	blister 60 unit(s)	1 mg	0.06	N05BA12	1 mg	60
	Bromazepam	pill	blister 40 unit(s)	1.5 mg	0.06	N05BA08	10 mg	6
	Bromazepam	pill	blister 60 unit(s)	1.5 mg	0.09	N05BA08	10 mg	9
	Bromazepam	pill	blister 40 unit(s)	3 mg	0.12	N05BA08	10 mg	12
	Bromazepam	pill	blister 60 unit(s)	3 mg	0.18	N05BA08	10 mg	18
	Bromazepam	pill	blister 60 unit(s)	6 mg	0.36	N05BA08	10 mg	36
	Bromazepam	pill	blister 40 unit(s)	6 mg	0.24	N05BA08	10 mg	24
	Bromazepam	capsule	blister 60 unit(s)	3 mg	0.18	N05BA08	10 mg	18
	Bromazepam	capsule	blister 60 unit(s)	1.5 mg	0.09	N05BA08	10 mg	9
	Bromazepam	pill	blister 20 unit(s)	3 mg	0.06	N05BA08	10 mg	6
	Bromazepam	pill	blister 20 unit(s)	6 mg	0.12	N05BA08	10 mg	12
	Bromazepam	capsule	blister 20 unit(s)	1.5 mg	0.03	N05BA08	10 mg	3
	Bromazepam	pill	blister 20 unit(s)	1.5 mg	0.03	N05BA08	10 mg	3
	Bromazepam	capsule	blister 20 unit(s)	3 mg	0.06	N05BA08	10 mg	6
	Brotizolam	pill	blister 14 unit(s)	0.25 mg	0.0035	N05CD09	0.25 mg	14
	Cetazolam	capsule	bottle 60 unit(s)	15 mg	0.9	N05BA10	30 mg	30
	Cetazolam	capsule	bottle 60 unit(s)	30 mg	1.8	N05BA10	30 mg	60
	Cetazolam	capsule	bottle 20 unit(s)	30 mg	0.6	N05BA10	30 mg	20
	Cetazolam	capsule	bottle 20 unit(s)	15 mg	0.3	N05BA10	30 mg	10
	Clobazam	pill	blister 30 unit(s)	10 mg	0.3	N05BA09	20 mg	15
	Clobazam	pill	blister 30 unit(s)	20 mg	0.6	N05BA09	20 mg	30
	Clobazam	pill	blister 20 unit(s)	20 mg	0.4	N05BA09	20 mg	20
	Clobazam	pill	blister 20 unit(s)	10 mg	0.2	N05BA09	20 mg	10
	Dip. chlorazepate	capsule	blister 60 unit(s)	10 mg	0.6	N05BA05	20 mg	30
	Dip. chlorazepate	capsule	blister 60 unit(s)	5 mg	0.3	N05BA05	20 mg	15
	Dip. chlorazepate	capsule	blister 60 unit(s)	15 mg	0.9	N05BA05	20 mg	45
	Dip. chlorazepate	capsule	blister 20 unit(s)	10 mg	0.2	N05BA05	20 mg	10
	Dip. chlorazepate	capsule	blister 20 unit(s)	5 mg	0.1	N05BA05	20 mg	5
	Dip. chlorazepate	capsule	blister 20 unit(s)	15 mg	0.3	N05BA05	20 mg	15
	Chlorodiazepoxide	coated tablet	blister 60 unit(s)	5 mg	0.3	N05BA02	30 mg	10
	Chlorodiazepoxide	coated tablet	blister 60 unit(s)	10 mg	0.6	N05BA02	30 mg	20
	Chlorodiazepoxide	coated tablet	blister 20 unit(s)	5 mg	0.1	N05BA02	30 mg	3.33
	Cloxazolam	pill	blister 60 unit(s)	2 mg	0.12	N05BA22	6 mg	20
	Cloxazolam	pill	blister 60 unit(s)	1 mg	0.06	N05BA22	6 mg	10
	Cloxazolam	pill	blister 20 unit(s)	2 mg	0.04	N05BA22	6 mg	6.67
	Cloxazolam	pill	blister 20 unit(s)	1 mg	0.02	N05BA22	6 mg	3.33
	Diazepam	pill	blister 60 unit(s)	5 mg	0.3	N05BA01	10 mg	30
	Diazepam	pill	blister 25 unit(s)	10 mg	0.25	N05BA01	10 mg	25
	Diazepam	pill	blister 60 unit(s)	10 mg	0.6	N05BA01	10 mg	60
	Diazepam	pill	blister 20 unit(s)	5 mg	0.1	N05BA01	10 mg	10
	Diazepam	pill	blister 25 unit(s)	5 mg	0.125	N05BA01	10 mg	12.5
	Diazepam	capsule	blister 60 unit(s)	6 mg	0.36	N05BA01	10 mg	36
	Diazepam	pill	blister 20 unit(s)	10 mg	0.2	N05BA01	10 mg	20
	Diazepam	extended release capsule	blister 60 unit(s)	10 mg	0.6	N05BA01	10 mg	60
	Diazepam	pill	blister 40 unit(s)	5 mg	0.2	N05BA01	10 mg	20
	Diazepam	extended release capsule	blister 20 unit(s)	10 mg	0.2	N05BA01	10 mg	20
	Diazepam	pill	blister 40 unit(s)	10 mg	0.4	N05BA01	10 mg	40
	Diazepam	capsule	blister 60 unit(s)	3 mg	0.18	N05BA01	10 mg	18
	Diazepam	capsule	blister 20 unit(s)	3 mg	0.06	N05BA01	10 mg	6
	Diazepam	rectal solution	cannula 5 unit(s) 2.5 ml	5 mg/2.5 ml	0.025	N05BA01	10 mg	2.5
	Diazepam	rectal solution	cannula 5 unit(s) 2.5 ml	10mg/2.5ml	0.05	N05BA01	10 mg	5
	Diazepam	capsule	blister 20 unit(s)	6 mg	0.12	N05BA01	10 mg	12
	Diazepam	rectal solution	unidosage 4 unit(s) 2.5 ml	5 mg/2.5 ml	0.02	N05BA01	10 mg	2
	Estazolam	pill	blister 14 unit(s)	2 mg	0.028	N05CD04	3 mg	9.33
	Estazolam	pill	blister 28 unit(s)	2 mg	0.056	N05CD04	3 mg	18.67
	Flurazepam	capsule	blister 20 unit(s)	30 mg	0.6	N05CD01	30 mg	20
	Flurazepam	capsule	blister 20 unit(s)	15 mg	0.3	N05CD01	30 mg	10
	Flurazepam	capsule	blister 14 unit(s)	30 mg	0.42	N05CD01	30 mg	14
	Loprazolam	pill	blister 15 unit(s)	1 mg	0.015	N05CD11	1 mg	15
	Lorazepam	pill	blister 60 unit(s)	2.5 mg	0.15	N05BA06	2,5 mg	60
	Lorazepam	pill	blister 30 unit(s)	1 mg	0.03	N05BA06	2,5 mg	12
	Lorazepam	pill	blister 60 unit(s)	1 mg	0.06	N05BA06	2,5 mg	24
	Lorazepam	pill	blister 30 unit(s)	2.5 mg	0.075	N05BA06	2,5 mg	30
	Lorazepam	pill	bottle 60 unit(s)	5 mg	0.3	N05BA06	2,5 mg	120
	Lorazepam	pill	blister 40 unit(s)	2.5 mg	0.1	N05BA06	2,5 mg	40
	Lorazepam	pill	blister 20 unit(s)	2.5 mg	0.05	N05BA06	2,5 mg	20
	Lorazepam	pill	blister 20 unit(s)	1 mg	0.02	N05BA06	2,5 mg	8
	Lorazepam	pill	blister 40 unit(s)	1 mg	0.04	N05BA06	2,5 mg	16
	Lorazepam	pill	bottle 20 unit(s)	5 mg	0.1	N05BA06	2,5 mg	40
	Mexazolam	pill	blister 60 unit(s)	1 mg	0.06	N05BA25	3 mg	20
	Mexazolam	pill	blister 20 unit(s)	1 mg	0.02	N05BA25	3 mg	6.67
	Midazolam	coated tablet	blister 20 unit(s)	15 mg	0.3	N05CD08	15 mg	20
	Midazolam	coated tablet	blister 14 unit(s)	15 mg	0.21	N05CD08	15 mg	14
	Oxazepam	pill	blister 30 unit(s)	15 mg	0.45	N05BA04	50 mg	9
	Oxazepam	pill	blister 30 unit(s)	50 mg	1.5	N05BA04	50 mg	30
	Prazepam	pill	blister 60 unit(s)	10 mg	0.6	N05BA11	30 mg	20
	Prazepam	pill	blister 20 unit(s)	10 mg	0.2	N05BA11	30 mg	6.67
	Temazepam	soft capsule	blister 14 unit(s)	20 mg	0.28	N05CD07	20 mg	14
	Triazolam	pill	blister 20 unit(s)	0.25 mg	0.005	N05CD05	0.25 mg	20
